# Epigenetic deregulation of GATA3 in neuroblastoma is associated with increased GATA3 protein expression and with poor outcomes

**DOI:** 10.1038/s41598-019-55382-6

**Published:** 2019-12-12

**Authors:** Bader Almutairi, Jessica Charlet, Anthony R. Dallosso, Marianna Szemes, Heather C. Etchevers, Karim T. A. Malik, Keith W. Brown

**Affiliations:** 1 University of Bristol, School of Cellular and Molecular Medicine, Biomedical Sciences Building, University Walk, Bristol, BS8 1TD UK; 2 Aix Marseille Univ, MMG, INSERM, U1251 Marseille, France; 3 Present Address: Zoology Department, College of Science, King Saud University, Riyadh, Kingdom of Saudi Arabia; 4 Present Address: Bayer - North American Headquarters, 100 Bayer Blvd, Hanover, NJ 07981 USA; 5 Present Address: Bristol Genetics Laboratory, Pathology Sciences, Southmead Hospital, Westbury-on-Trym, Bristol, BS10 5NB UK

**Keywords:** Cancer, Paediatric cancer

## Abstract

To discover epigenetic changes that may underly neuroblastoma pathogenesis, we identified differentially methylated genes in neuroblastoma cells compared to neural crest cells, the presumptive precursors cells for neuroblastoma, by using genome-wide DNA methylation analysis. We previously described genes that were hypermethylated in neuroblastoma; in this paper we report on 67 hypomethylated genes, which were filtered to select genes that showed transcriptional over-expression and an association with poor prognosis in neuroblastoma, highlighting *GATA3* for detailed studies. Specific methylation assays confirmed the hypomethylation of *GATA3* in neuroblastoma, which correlated with high expression at both the RNA and protein level. Demethylation with azacytidine in cultured sympathetic ganglia cells led to increased *GATA3* expression, suggesting a mechanistic link between *GATA3* expression and DNA methylation. Neuroblastomas that had completely absent *GATA3* methylation and/or very high levels of protein expression, were associated with poor prognosis. Knock-down of *GATA3* in neuroblastoma cells lines inhibited cell proliferation and increased apoptosis but had no effect on cellular differentiation. These results identify *GATA3* as an epigenetically regulated component of the neuroblastoma transcriptional control network, that is essential for neuroblastoma proliferation. This suggests that the *GATA3* transcriptional network is a promising target for novel neuroblastoma therapies.

## Introduction

Neuroblastoma (NB) is one of the commonest extra-cranial solid malignancies of childhood, which arises as a result of disordered development of the sympathetic nervous system from neural crest cells^1,2^. Neuroblastoma is clinically heterogeneous, with younger patients (<18months) generally having localised tumours and good outcomes, whereas older children (>18months) mostly have disseminated tumours at diagnosis and poor outcomes^3^.

The clinical heterogeneity of neuroblastoma is reflected in its molecular pathogenesis, where no single pathway has been identified as being critical for tumour development. Oncogene activations were the first genetic alterations identified in neuroblastoma; initially *MYCN* amplification was identified in high-risk tumours^4^ and later ALK mutations were discovered in inherited neuroblastoma and some sporadic high-risk tumours^5,6^. Mutations in tumour suppressor genes such as *PHOX2B*^7^ and *NF1*^8^ have also been reported. Recent genome-wide analyses have identified genomic mutations and other alterations in chromatin remodelling genes such as *ATRX*, *ARID1A* and *ARID1B*, in components of the *RAC-RHO* pathway^9–11^ and in *TERT*^12,13^, with relapsed tumours demonstrating an increased mutation rate^14,15^.

Like most childhood cancers, neuroblastomas contain fewer mutations than adult cancers^16,17^, with some tumours apparently containing no detectable driver mutations^9–11^. In low-risk neuroblastomas, copy-number changes may drive tumorigenesis^18^, but the lack of driver mutations in many cases, emphasises the need to consider other mechanisms of pathogenesis, such as epigenetic alterations^19^.

Epigenetic changes have been shown to play an important role in neuroblastoma, with well-characterised examples of silencing of tumour suppressor genes by DNA methylation^20–24^, or by repressive histone modifications^24–26^. Recent genome-wide studies have characterised widespread alterations in DNA methylation in neuroblastoma^24,27–34^, confirming previous associations between a hypermethylator phenotype and poor prognosis, as well as identifying subgroup-specific epigenetic profiles^35^.

In order to identify epigenetic changes associated with the development of neuroblastoma, we previously used genome-wide DNA methylation analysis to compare neuroblastoma cells to neural crest cells, their cellular precursors, and identified *MEGF10* as an epigenetically repressed putative tumour suppressor gene^24^. Interestingly, like other recent studies^31,32^, we found a preponderance of hypomethylated genes, suggesting that epigenetic activation of normally silenced genes in neural crest cells is critical in neuroblastoma pathogenesis. In this paper we have therefore investigated the hypomethylated genes identified in our previous work^24^, demonstrating that *GATA3*, a transcription factor known to be critical in development of the sympathetic nervous system^36–38^ and a gene often dysregulated in diverse human cancers^39^, is frequently affected by epigenetic deregulation in neuroblastoma.

## Results

### Hypomethylated genes in neuroblastoma

We have previously described our genome-wide analysis of DNA methylation in neuroblastoma, in which we used methyl CpG immunoprecipitation (MCIP) and promoter microarrays, to compare neuroblastoma cell lines with human neural crest cells (hNCC)^24^. In our previous paper, we described our results on genes that were hypermethylated in neuroblastoma compared to hNCC^24^; in this paper we discuss hypomethylated genes.

**Figure 1:**
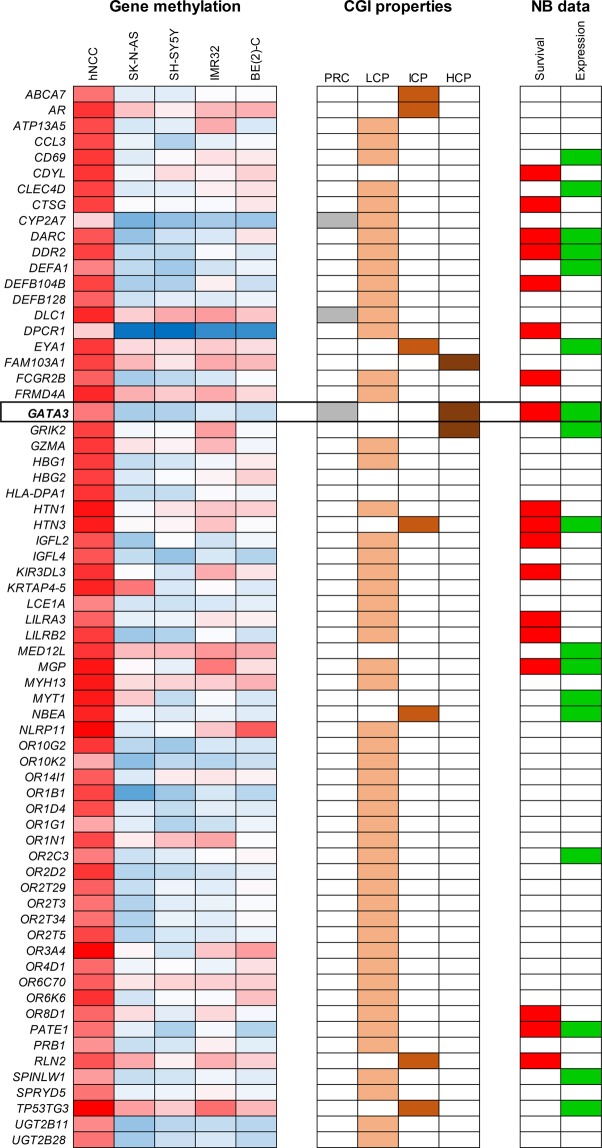
Hypomethylated genes in neuroblastoma. Genes identified by MCIP as hypomethylated in four neuroblastoma cell lines compared to hNCC. The first five columns (“Gene methylation”) are a heatmap of gene methylation values (blue = low, red = high). CGI (CpG island) properties: PRC shows genes that are polycomb marked in ES cells, LCP, ICP and HCP define which promoter CGIs have low, intermediate or high CpG content. For quantitative DNA methylation results and further explanation of PRC, LCP, ICP and HCP, see Table S1. NB data: “Survival” shows genes whose increased expression is significantly associated with reduced relapse-free survival in neuroblastoma (p < 0.05, log rank test); data generated in R2 using GSE16476. “Expression” shows genes whose RNA expression is increased in both neuroblastoma cell lines (GSE28019) and neuroblastoma tumours (GSE16476) compared to neural crest cells (GSE14340); comparison was made using the “Megasampler” function in R2 Genomics Analysis and Visualization Platform (http://r2.amc.nl). See Table S1 for full results.

A total of 67 genes were found by MCIP to be hypomethylated in neuroblastoma cell lines compared to hNCC (Fig. 1 and Supplementary Table S1). Gene ontology analysis showed this group to be enriched in genes regulating biological processes, G-protein coupled receptor signalling and sensory perception (Supplementary Table S2). To filter the hypomethylated genes for those likely to be functionally important in neuroblastoma pathogenesis, we used publicly available databases to search for genes that; (1) were overexpressed in neuroblastoma cell lines and tumour tissue compared to hNCC (reasoning that hypomethylated genes would be expected to have elevated expression) and (2) showed an association of high gene expression in tumours with poorer patient survival (to identify genes affecting neuroblastoma biological properties). Six genes had both the required over-expression and association with poor patient survival, but only one, *GATA3*, had a high CpG-content CpG island (CGI), suggesting it might be most susceptible to epigenetic deregulation (Fig. 1 and Supplementary Table S1). In addition, *GATA3* was known to be of importance in development of sympathetic nervous tissue, from which neuroblastoma derives^1,2^. We therefore went on to examine the DNA methylation, expression and functional relevance of *GATA3* in neuroblastoma.

### GATA3 methylation in neuroblastoma

**Figure 2:**
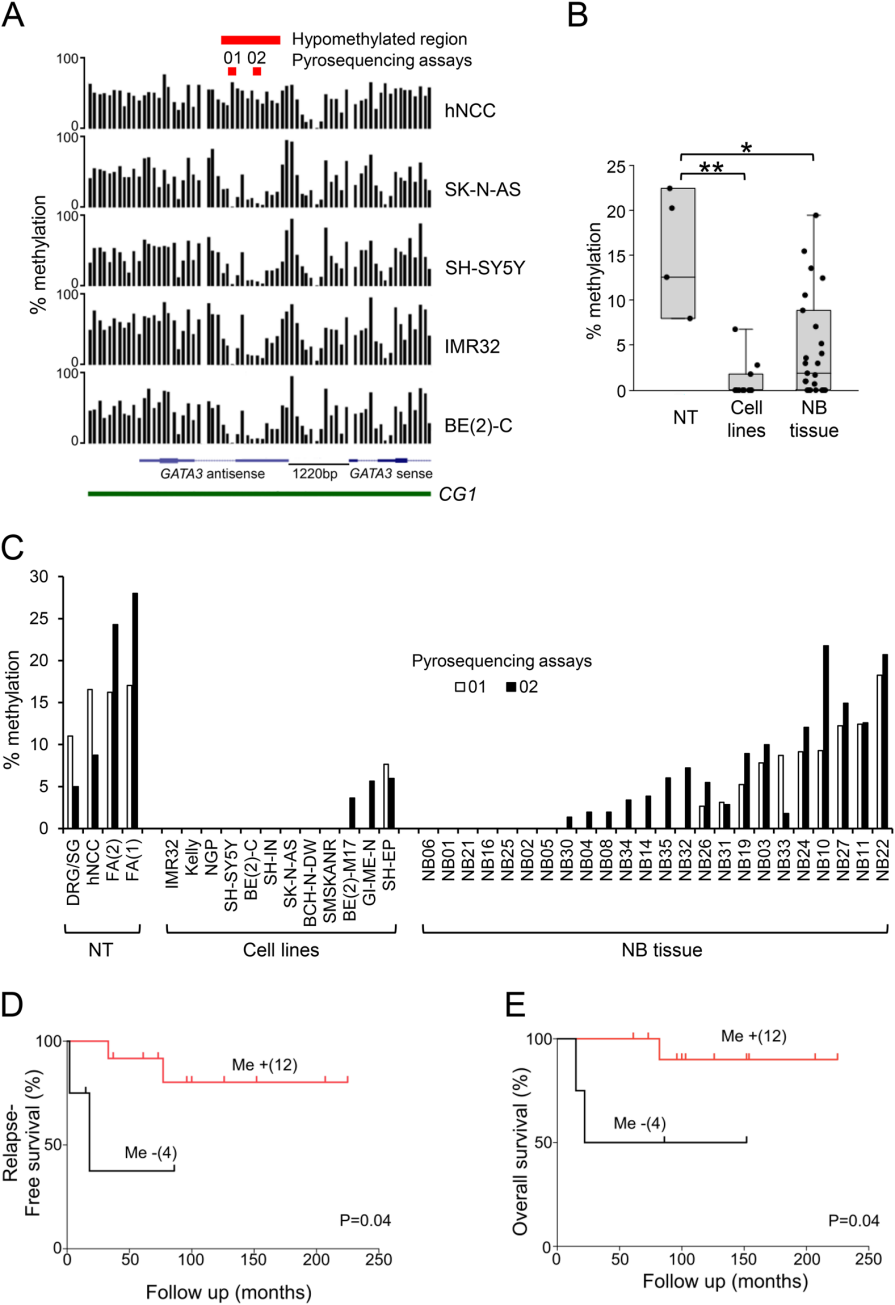
*GATA3* DNA methylation in neuroblastoma. (**A**) *GATA3* DNA methylation detected by MCIP. Black bars show the probe ratios derived from MCIP for hNCC and four neuroblastoma cell lines, positioned on the *GATA3* CpG island promoter region, showing the sense and antisense transcripts and CpG island (CGI) (human genome build NCBI36/Hg18 visualised on the UCSC genome browser; http://genome.ucsc.edu). The positions of the hypomethylated region and the two pyrosequencing assays (01 and 02) are shown in red at the top. (**B**) Dotboxplot of *GATA3* antisense DNA methylation measured by pyrosequencing in normal tissues (NT, n = 4), neuroblastoma cell lines (Cell lines, n = 12), and neuroblastoma tumour tissue (NB tissue, n = 24), using the average of pyrosequencing assays 01 and 02; full results in C; *p < 0.05, **p < 0.005, Bonferroni corrected Mann-Whitney test. (**C**) DNA methylation in the *GATA3* antisense region in normal tissues (NT), NB cell lines (Cell lines) and NB tumour tissue (NB tissue), using pyrosequencing assays 01 (unfilled bars) and 02 (filled bars). The genomic positions of assays 01 and 02 are shown in part A. (**D**,**E**) Kaplan–Meier survival curves (D, relapse-free survival; E, overall survival) taken from the dataset of NB patients in B and C for whom survival data were available. Me-, tumours with no *GATA3* DNA methylation; Me + tumours with DNA methylation (using the average of pyrosequencing assays 01 and 02). p values from log-rank test.

Examination of the MCIP data showed that the major area of hypomethylation in neuroblastoma cell lines compared to hNCC, centred on the start of the *GATA3* antisense transcripts in the CGI (Fig. 2A). We used two commercially-available pyrosequencing assays to examine DNA methylation in this region (Fig. 2A) and found that neuroblastoma cell lines and tumour tissue were significantly hypomethylated compared to a panel of control tissues, consisting of hNCC, fetal adrenal tissue (FA) and dorsal root ganglia/sympathetic ganglia cell lines (DRG/SG) (Fig. 2B,C).

We extracted *GATA3* DNA methylation data from the publicly available dataset GSE54719, which confirmed hypomethylation across the start of the *GATA3* antisense transcripts in neuroblastoma tumours, compared to adrenal tissue (Supplementary Fig. S1).

Using survival data available for our patient cohort, we demonstrated that complete absence of DNA methylation within the hypomethylated region was associated with worse relapse-free survival and worse overall survival in neuroblastoma patients (Fig. 2D,E).

These results suggested that epigenetic deregulation of *GATA3* by altered DNA methylation might be functionally important in neuroblastoma. We therefore investigated whether DNA methylation affected *GATA3* expression in neuroblastoma.

### Relationship between GATA3 methylation and expression

**Figure 3:**
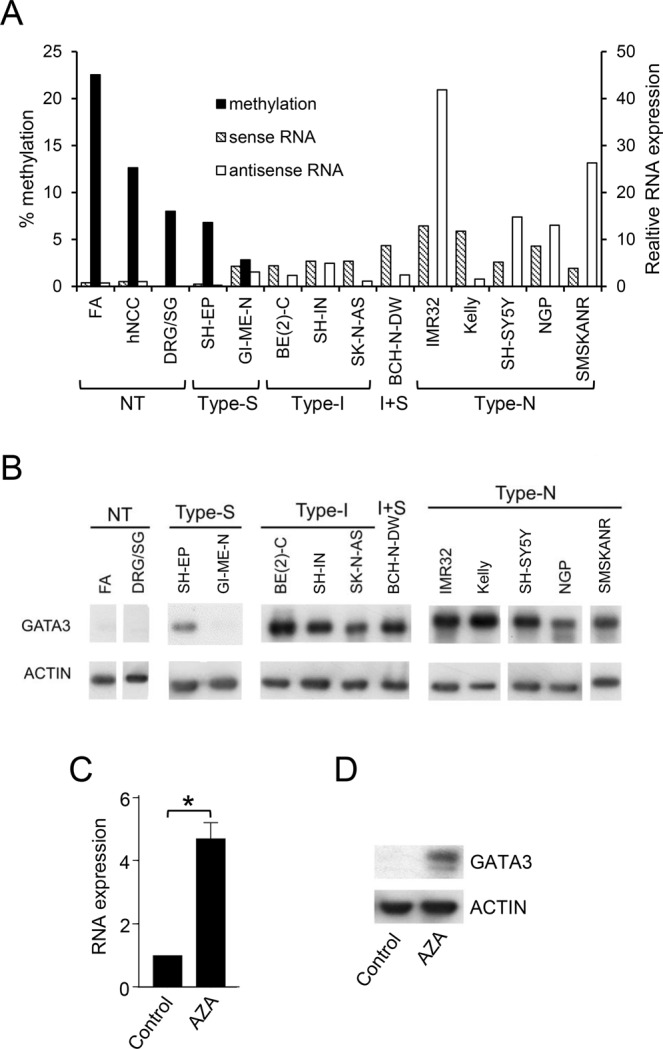
*GATA3* DNA methylation and expression. (**A**) *GATA3* sense (hatched bars) and antisense (unfilled bars) RNA expression assayed by QPCR, and DNA methylation levels (black bars) detected by pyrosequencing, in control tissues and neuroblastoma cell lines. RNA levels were normalized to endogenous levels of *TBP* and expressed relative to hNCC. DNA methylation was calculated as the average of the 01 and 02 pyrosequencing assays. (**B**) GATA3 protein levels assayed by Western blot in normal tissues (NT) and type-S, type-I, I + S and type-N neuroblastoma cell lines, with ACTIN as a loading control. Uncropped blots are shown in Supplementary Fig. S10. (**C**) *GATA3* sense RNA expression in DRG/SG cells treated with 2 μM AZA for 6 days. RNA levels were normalized to endogenous levels of *TBP* and expressed relative to control. Mean ± S.E.M of three experiments; *p < 0.05, paired t test. (**D**) Western Blot of GATA3 protein expression in in DRG/SG cells treated with 2 μM AZA for 6 days, with ACTIN as a loading control. Uncropped blots are shown in Supplementary Fig. S10.

In almost all neuroblastoma cell lines, there was little or no *GATA3* DNA methylation, and much higher levels of expression of both the sense and antisense *GATA3* RNAs, compared to normal tissues (FA, hNCC and DRG/SG), which had 7–22% DNA methylation, and very low RNA expression (Fig. 3A). The exceptions were SH-EP and GI-ME-N, S-type neuroblastoma cell lines (Supplementary Table S3), which had some *GATA3* methylation and low RNA expression (Fig. 3A). In general, *GATA3* sense and antisense RNA levels correlated positively, with N-type neuroblastoma cells generally having the highest antisense expression (Fig. 3A and Supplementary Fig. S2C). There was an inverse correlation between *GATA3* DNA methylation and RNA expression in normal and neuroblastoma cell lines (Supplementary Fig. S2A,B).

GATA3 protein was overexpressed in nearly all neuroblastoma cell lines compared to normal tissue (Fig. 3B). The S-type cell line GI-ME-N had undetectable GATA3 protein and SH-EP had one of the lowest levels of expression (Fig. 3B), which agrees with the low levels of *GATA3* RNA expression detected in these two S-type cell lines (Fig. 3A). Overall, there was a good correlation between *GATA3* RNA and protein expression in the neuroblastoma cell lines (Supplementary Fig. S2D).

These findings suggested a possible relationship between *GATA3* DNA methylation and expression, which we tested by treating DRG/SG cells with the DNA methylation inhibitor 5-aza-2′-deoxycytidine (AZA). AZA treatment resulted in a five-fold increase in *GATA3* RNA expression, as well as increased expression of GATA3 protein (Fig. 3C,D), implying that DNA methylation plays a mechanistic role in regulating *GATA3* expression in sympathetic nervous system-derived cells. We then proceeded to investigate *GATA3* RNA and protein expression in neuroblastoma tumour tissues from different stages, to search for any relationship between *in vivo* biological properties of tumours and *GATA3* expression.

### GATA3 RNA and protein expression in neuroblastoma

**Figure 4:**
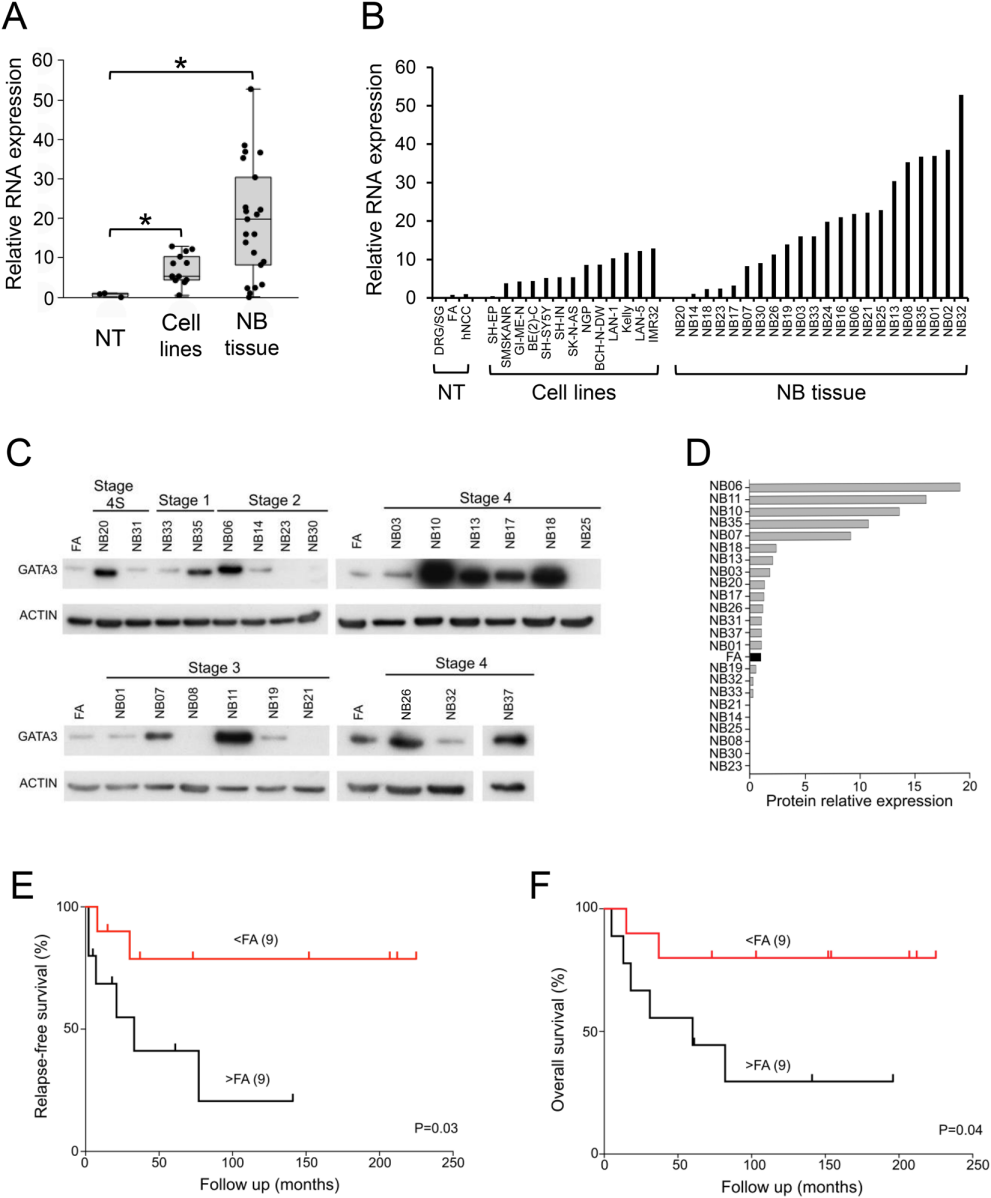
*GATA3* RNA and protein expression in neuroblastoma. (**A**) Dotboxplot of *GATA3* sense RNA expression assayed by QPCR in normal tissues (NT, n = 3), neuroblastoma cell lines (Cell lines, n = 13), and neuroblastoma tumour tissue (NB tissue, n = 22). RNA levels were normalized to endogenous levels of *TBP* and expressed relative to hNCC; full results in B; *p < 0.05, Bonferroni corrected Mann-Whitney test. (**B**) Individual *GATA3* RNA expression by QPCR. (**C**) GATA3 protein levels assayed by Western blot in stage 1, 2, 3, 4 and 4S neuroblastoma tumour tissue, with ACTIN as a loading control. Uncropped blots are shown in Supplementary Fig. S11. (**D**) GATA3 protein levels in neuroblastoma tumour tissue relative to ACTIN (average of two experiments), expressed as a ratio of the level in fetal adrenal (FA; black bar). (**E**,**F**) Kaplan–Meier survival curves (**E)**, relapse-free survival; (**F)** overall survival) taken from the dataset of NB patients in C and D for whom survival data were available. >FA, tumours with high level of GATA3 protein expression (greater than FA); <FA, NB patients with low level of GATA3 protein expression (less than FA). p values from log-rank test.

*GATA3* sense RNA expression was significantly elevated in neuroblastoma cell lines and tumours compared to normal tissues (FA, hNCC and DRG/SG; Fig. 4A,B). These results were replicated in publicly available gene expression data (Supplementary Fig. S3A), which also showed that high *GATA3* RNA expression was associated with poor overall survival in neuroblastoma (Supplementary Fig. S3B).

Almost all neuroblastoma tumours expressed detectable GATA3 protein, of which 61% expressed higher levels than FA (Fig. 4C,D). In those tumours for which survival data were available, relapse-free survival and overall survival were both worse in patients whose tumours expressed more GATA3 protein than FA (Fig. 4E,F).

These results suggested that expression levels of *GATA3* were correlated with the clinical properties of neuroblastoma tumours, so we examined GATA3 effects on cell proliferation and death, which could provide an explanation for these correlations.

### Effects of GATA3 silencing in neuroblastoma cell lines

**Figure 5:**
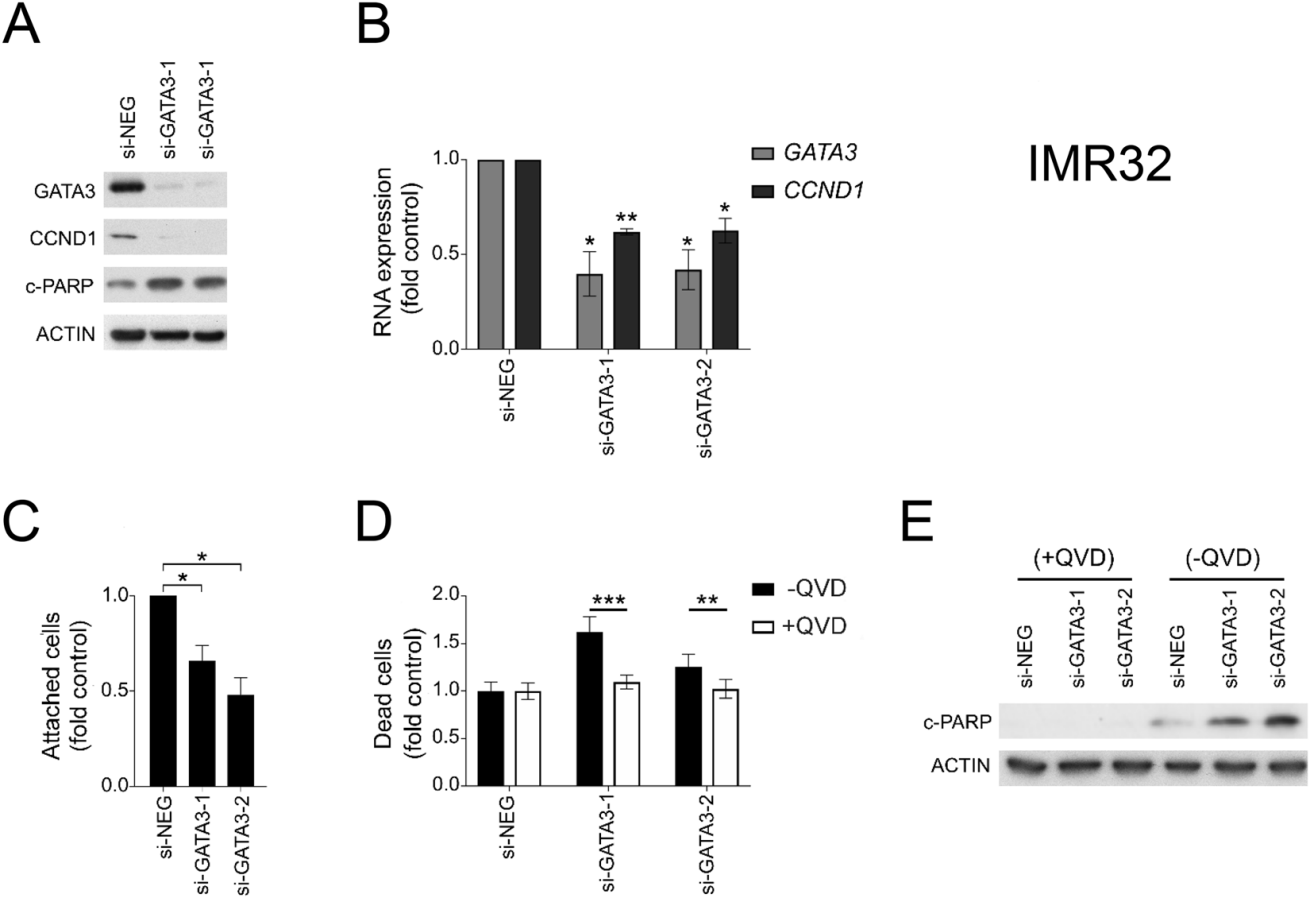
GATA3 biological functions. Growth of IMR32 neuroblastoma cell line after transfection with non-targeting siRNA (siNEG), or *GATA3* siRNAs (si-GATA3-1 and si-GATA3-2). (**A**) Western Blot of GATA3, cleaved PARP (c-PARP) and CCND1 protein expression (representative of three experiments), with ACTIN as a loading control. Uncropped blots are shown in Supplementary Fig. S12. (**B**) *GATA3* and *CCND1* RNA expression assayed by QPCR expressed relative to siNEG controls. Mean ± S.E.M of three experiments, *p < 0.05; **p < 0.01, paired t test. (**C**) Attached cell counts expressed relative to siNEG controls. Mean ± S.E.M of three experiments, *p < 0.05, paired t test. (**D**) Dead cell counts (floating cells that were trypan blue permeable) expressed relative to siNEG control in cells treated caspase 3 inhibitor QVD (+QVD, unfilled bars) or with drug solvent (DMSO; -QVD, filled bars). Mean ± S.E.M of three experiments, **p < 0.01, **p < 0.001, t test. (**E**) Western Blot of c-PARP protein expression in transfected cells with (+QVD) or without (-QVD) QVD- treatment (representative of three experiments), with ACTIN as a loading control. Uncropped blots are shown in Supplementary Fig. S12.

IMR32, Kelly and NGP neuroblastoma cell lines, all of which expressed high levels of *GATA3* sense RNA and GATA3 protein (Fig. 3A,B), were transfected with siRNAs to knock-down *GATA3* expression, and then cell proliferation and cell death were assessed by a variety of assays (Fig. 5, Supplementary Fig. S4). In all cell lines, the *GATA3* siRNAs were effective in down regulating both *GATA3* RNA expression and GATA3 protein expression (Fig. 5A,B, Supplementary Fig. S4A,B,F,G). *GATA3* knock-down inhibited cell proliferation (Fig. 5C, Supplementary Fig. S4C,H) and increased the number of dead cells in the cultures (Fig. 5D, Supplementary Fig. S4D,I). Cleaved PARP (c-PARP) protein levels increased in *GATA3* siRNA-treated cells (Fig. 5A, Supplementary Fig. S4A,F), and this increase was abrogated by treating cells with the caspase inhibitor quinolyl-valyl-O-methylaspartyl-[2,6-difluorophenoxy]-methyl ketone (QVD) (Fig. 5E, Supplementary Fig. S4E,J). QVD treatment also abrogated the increased dead cell counts after *GATA3* knock-down (Fig. 5D, Supplementary Fig. S4D,I), which together with PARP cleavage, showed that the cell death was caused by caspase-mediated apoptosis.

To further investigate the possible pathways by which *GATA3* influences neuroblastoma proliferation, we examined cyclin D1 (CCND1), one of the known targets of *GATA3* that is involved in cell cycle regulation^40^. Flow cytometry demonstrated that in almost all *GATA3* siRNA knock-downs, there was a reduced proportion of cells in G2-M, consistent with disrupted cell cycle control (Supplementary Fig. S5). Knock-down of GATA3 in neuroblastoma cell lines reduced CCND1 protein and RNA expression substantially (Fig. 5A,B, Supplementary Fig. S4A,B,F,G). In neuroblastoma tumours, most showed increased expression of CCND1 compared to FA, where it was undetectable (Supplementary Fig. S6A) and there was a good correlation between GATA3 protein expression and CCND1 protein expression (Supplementary Fig. S6B). This suggested that one of major mechanisms by which GATA3 affects neuroblastoma cell proliferation may be via modulating CCND1 levels.

To assess whether GATA3 knock-down might be affecting cellular differentiation, we investigated the expression of a range of differentiation markers in the siRNA-transfected neuroblastoma cell lines (Supplementary Fig. S7). We assayed markers of both mesenchymal differentiation (*VIM*, *PRRX1* and *SNAI1*) and adrenergic differentiation (*NCAM*, *NF68*), but observed no reproducible changes in gene expression, suggesting that GATA3 knock-down only affects proliferation and death, but not differentiation, in neuroblastoma cells.

## Discussion

In this paper we have examined genes that were hypomethylated in neuroblastoma, as identified by our previous genome-wide analysis of DNA methylation, comparing hNCC to neuroblastoma cell lines^24^. We have now shown that *GATA3*, an important regulator of sympathetic nervous system development^36–38^, is hypomethylated in neuroblastoma cell lines and tumour tissues (Figs. 2B,C, 3A and Supplementary Fig. S1). Hypomethylation of *GATA3* correlated with upregulated expression of both *GATA3* RNA and GATA3 protein (Figs. 3A,B, 4A–D and Supplementary Fig. S3).

These results suggest that the development of neuroblastoma from neural crest derivatives involves a change in the epigenetic state of *GATA3*, that is controlled at least partially by DNA methylation, which is known to be an important factor in neural crest development^41^. Interestingly, we found a maximum of 22% GATA3 DNA methylation in the normal tissues that we examined (Figs. 2C and 3A), suggesting that only a subpopulation of these cells have DNA methylated at *GATA3*, which could reflect an intrinsic heterogeneity in the neural crest population^42^.

The inverse correlation between *GATA3* DNA methylation and RNA expression (Fig. 3A and Supplementary Fig. S2A,B), implied a functional role for DNA methylation in the repression of *GATA3* transcription, which was supported by the increase in *GATA3* expression following AZA treatment of DRG/SG cells (Fig. 3C,D). The five-fold increase in *GATA3* RNA expression was perhaps surprising, considering the relatively low levels of *GATA3* DNA methylation in DRG/SG cells (Fig. 2C). This could suggest that there is a small subpopulation of methylated cells, which show greatly elevated *GATA3* expression when they become demethylated, or alternatively that the transcriptional regulation of *GATA3* may be via another epigenetically regulated factor, rather than by a direct epigenetic effect on *GATA3* itself.

Others have reported DNA methylation at the *GATA3* CGI in breast cancer^43,44^ bladder cancer^45^ and some leukaemias^46^, with direct evidence that DNA methylation regulates *GATA3* expression^46,47^. In our results, *GATA3* methylation was confined to the start of the *GATA3* antisense transcript (Fig. 2A), although AZA treatment upregulated *GATA3* sense RNA and GATA3 protein in methylated DRG/SG cells (Fig. 3C,D), suggesting a possible role for the antisense RNA in regulating *GATA3* sense expression. We found a good correlation between *GATA3* sense and antisense expression (Supplementary Fig. S2C), in agreement with a previous report^48^. That report suggested that the proximity of the transcriptional start sites of the *GATA3* sense and antisense RNAs could imply the presence of a bidirectional promoter^48^. However, it has recently been shown that the *GATA3* antisense RNA can bring MLL to the *GATA3* promoter, to increase permissive chromatin marks and thus directly upregulate *GATA3* expression^49^. Thus, it is likely that demethylation of *GATA3* in neuroblastoma has an indirect effect, by increasing *GATA3* antisense expression, which in turn attracts positive epigenetic regulators to the *GATA3* promoter region.

Our siRNA experiments demonstrated that knock-down of GATA3 expression reduced cell proliferation and increased apoptosis in neuroblastoma cell lines (Fig. 5, Supplementary Fig. S4). These results agree with previous studies that have shown an essential role for GATA3 in the proliferation of neuroblastoma cells *in vitro* and for tumorigenicity *in vivo*^50,51^. We found that knock-down of GATA3 decreased CCND1 expression in neuroblastoma cell lines (Fig. 5, Supplementary Fig. S4) and that in tumour samples, there was high expression of CCND1 compared to FA and a good correlation between GATA3 and CCND1 expression (Supplementary Fig. S6), as found by others^40,52,53^. Thus, one of the likeliest mechanisms by which GATA3 affects cell proliferation in neuroblastoma, is via regulation of its transcriptional target cyclin CCND1^40,54^, which when knocked-down, shows similar effects on cell proliferation to GATA3^53^. Given that we observed no effects on cellular differentiation when GATA3 was knocked-down (Supplementary Fig. S7), it seems likely that the major mechanism by which GATA3 affects neuroblastoma development may be via its effects on cellular proliferation and death (Fig. 5 and Supplementary Fig. S4), rather than by affecting cellular differentiation.

Two recent papers have delineated super-enhancer-associated transcriptional circuits that divide neuroblastoma cells into two distinct differentiation states^55,56^; corresponding to undifferentiated mesenchymal cells (MES), as found in hNCC, and a sympathetic adrenergic identity (ADRN), in which GATA3 plays a critical role as a master transcription factor^55,56^. Neuroblastomas contain mixtures of these two cell types, but with the majority being highly enriched in adrenergic cells, leading to high GATA3 expression, as we have observed (Fig. 4).

Our genome-wide DNA methylation study compared hNCC (mesenchymal differentiation) to neuroblastoma cell lines, which mostly have a predominately adrenergic state^55,56^. Hierarchical clustering of this data demonstrates the ability of DNA methylation to discriminate between cell lines representing these different lineages (Supplementary Fig. S8A). Interestingly, this analysis showed that SK-N-AS clusters separately from the three other neuroblastoma cell lines, which agrees with the super-enhancer profiling, which places SK-N-AS as being intermediate between the ADRN and mesenchymal MES signatures^55,56^.

We also showed that adrenergic (ADRN) signature genes were more highly methylated in hNCC than were mesenchymal (MES) signature genes^56^ (Supplementary Fig. S8B). Thus, distinct patterns of DNA methylation are associated with alternate differentiation states, suggesting that epigenetic modifications may play an important role in defining or stabilising cell identity in neuroblastoma.

Among neuroblastoma cell lines, the S-type cells GI-ME-N and SH-EP are in the mesenchymal differentiation pathway, like hNCC cell lines^55,56^. Interestingly, our results identified GI-ME-N and SH-EP as the only two neuroblastoma cell lines that had *GATA3* DNA methylation, like hNCC, with corresponding low levels of expression of GATA3 protein (Fig. 3). Therefore, our results identify DNA methylation of *GATA3* as a characteristic of the mesenchymal hNCC-like differentiation pathway, with loss of *GATA3* methylation in neuroblastoma cell lines and tumours, which contain mainly adrenergic cells. However, we found that knock-down of GATA3 did not alter the differentiation status of the three neuroblastoma cell lines tested (Supplementary Fig. S7). This suggests that inactivation of just one of the transcription factors defining the adrenergic phenotype, is insufficient to drive neuroblastoma cells towards the mesenchymal phenotype, presumably due to the functional redundancy within the complex regulatory network. Our results therefore show that GATA3 is essential for the sustained proliferation of adrenergic neuroblastoma cells, but not solely responsible for defining their lineage.

The cellular composition of neuroblastomas does not appear to correspond to clinical outcome directly, although epigenetic plasticity may allow interconversion between the cell types, driving drug resistance^55,56^. Others have previously shown a high level of *GATA3* expression in neuroblastoma^50,57^, and suggested that GATA3 is a useful distinguishing diagnostic marker for neuroblastoma^58^. Our results showed that a complete absence of *GATA3* DNA methylation (Fig. 2D,E) and a high level of GATA3 protein expression (Fig. 4E,F), were both indicators of poor prognosis in neuroblastoma. Thus, although the adrenergic composition of neuroblastomas may not in itself be sufficient to determine the clinical phenotype of neuroblastomas, we suggest that epigenetic defects in *GATA3* may deregulate the complex transcriptional networks controlling neuroblastoma and thus influence clinical outcomes. Therefore, the GATA3 transcriptional network is a promising target for novel neuroblastoma therapies.

## Materials and Methods

### Cell culture and neuroblastoma tumour samples

Cell lines (Supplementary Table S3) were obtained from ECACC, apart from BCH-N-DW, which is a novel neuroblastoma cell line derived from a bone marrow biopsy (kind gift from Dr C. McConville, unpublished data). Cell lines were cultured in DMEM/F12-HAM medium (Sigma) supplemented with 10% fetal bovine serum, 100U/ml penicillin, 0.1 mg/ml streptomycin, 2mM L-glutamine and 1% non-essential amino acids (Sigma) at 37 °C in a 5% CO_2_ incubator.

Human neural crest cell lines (hNCC) were cultured as described previously^59,60^. Dorsal root ganglia/sympathetic ganglia cell lines (DRG/SG) were derived from cells growing away from dorsal root and sympathetic ganglia with some proximal nerve fascicles still attached, explanted from normal 8.5 to 9.5 gestational week human embryos (French Agence de la Biomédecine authorization PSF14-011). Within 18 hours of being explanted, the remaining pieces of ganglionic tissue were removed manually, and the cells passed into a new culture dish and cultured thereafter in the same medium as used for human neural crest cells (hNCC)^60^ (Etchevers, unpublished). DRG/SG lines expressed sympathetic adrenergic markers *DBH* and *MYCN* to a higher level than lines derived from dorsal root ganglia alone (DRG; Supplementary Fig. S9).

Neuroblastoma tumour samples (Supplementary Table S4) were obtained from Bristol Children’s hospital with informed consent (from parent and/or legal guardian for children less than 18 years of age) and with appropriate ethical approval (E5797; South West – Central Bristol Research Ethics Committee (UK)). All methods were performed in accordance with the relevant guidelines and regulations, including those specified in the UK Human Tissue Act 2004.

### Cell counts

For cell viability and determining concentration, Trypan Blue stain (0.4%; Sigma) was mixed with cells in a ratio 1/1 before being counted by a Countess cell counter (Invitrogen). Dead cells were assayed by counting the number floating (unattached) cells that were trypan blue permeable.

### Flow cytometry and cell cycle analysis

Cells were removed from flasks with trypsin, pelleted by centrifugation and fixed in 70% ethanol at −20 °C for 24 hours. They were then treated with RNAase (Qiagen) and stained with 50 μg/ml Propidium iodide (Sigma) and incubated at 37 °C for 15 min in the dark. Stained cells were analysed using LSR Fortessa X20 (BD Biosciences) using FACS DIVA8 software (Becton-Dickinson Immunocytometry Systems) and at least 10,000 events were collected. Cells cycle analysis was performed using Flow Jo software V10.

### 5-Aza-2′-deoxycytidine treatment

Cell lines were incubated in medium containing 2 µM 5-aza-2′-deoxycytidine (AZA; Sigma) for up to 6 days, with a medium change every three days. Control cultures received equivalent volumes of drug solvent (DMSO).

### Quinolyl-valyl-O-methylaspartyl-[2,6-difluorophenoxy]-methyl ketone treatment

Cell lines were incubated in medium containing 10 µM quinolyl-valyl-O-methylaspartyl-[2,6-difluorophenoxy]-methyl ketone (QVD; Sigma) for up to three days. Control cultures received equivalent volumes of drug solvent (DMSO).

### GATA3 transient silencing

Cells were transfected with siRNAs (50 nM) against *GATA3*, or a non-targeting siRNA negative control (Supplementary Table S5; all synthesised by Sigma), using Lipofectamine 2000 transfection reagent (Invitrogen) and harvested after 72 hrs.

### Pyrosequencing assay of GATA3 DNA methylation

DNA was bisulfite converted (EZ DNA Methylation Gold kit; Zymo Research), amplified with biotinylated primers (Qiagen) using a Pyromark PCR kit (Qiagen) and pyrosequenced on a PyroMark Q96 instrument (Qiagen).

Pyrosequencing assay 01 for *GATA3* was PM00042273 (Qiagen); sequence analysed TTAATYGYGAGTATTAAGTYGGATTGGTYGGGGA, and assay 02 was PM00042280 (Qiagen); sequence analysed GATGTTTTTTAATTGGGTYGTTTAATAAYGGGA.

### RNA extraction, cDNA synthesis and RT-PCR

Total RNA was extracted with a RNeasy kit (Qiagen), DNase treated with TURBO DNA-free (Ambion) and cDNA synthesized using the Thermoscript RT-PCR system (Invitrogen). gene-specific primers (Supplementary Table S6) were used for QPCR (QuantiTect SYBR Green; Qiagen) on an MX3000P real-time PCR machine (Stratagene), normalising the amount of target gene to the endogenous level of *TBP*. Some assays were normalised relative to expression in Human universal RNA (Agilent).

### Protein extraction and western blotting

Cultured cells were washed with ice-cold PBS and lysed in cell lysis buffer (Cell Signaling), with complete mini inhibitors (Roche) for 10 min on ice, and then sonicated for 5 min at high intermittent pulses (30/30) (Diagenode, Bioruptor). Neuroblastoma tumour samples were homogenised in cell lysis buffer, then processed as for cultured cells. Samples were centrifuged for 10 min at 10,000 g at 4 °C to remove any cell debris and typically 25 µg proteins were separated on SDS-polyacrylamide gels and analysed by Western blotting. Fetal adrenal protein was from Biochain. Primary antibodies were against GATA3 (Rabbit monoclonal, Abcam Ab199428), CCND1 (Rabbit monoclonal, Cell Signaling), cleaved-PARP (c-PARP; Rabbit monoclonal, Abcam Ab32064), Beta ACTIN (Mouse monoclonal, Sigma A3854), followed by secondary HRP-labelled anti-rabbit (Sigma A6154) or HRP-labelled anti-mouse (Sigma A4416). Detection was with Lumiglo chemiluminescent substrate (KPL) and X-ray films were imaged on a flatbed scanner and analysed using Image J (http://imagej.nih.gov/ij/).

## Supplementary information

Supplementary data

## Data Availability

The methyl CpG immunoprecipitation (MCIP) data upon which this study is based, has previously been deposited in GEO; accession number GSE71958.
