# Supplementary material for: Epigenetic deregulation of GATA3 in neuroblastoma is associated with increased GATA3 protein expression and with poor outcomes
Source: Sci Rep. 2019 Dec 12;9:18934. doi: 10.1038/s41598-019-55382-6 (PMC6908619; doi:10.1038/s41598-019-55382-6)
Supplement: Supplementary file 1 — Supplementary data [file 41598_2019_55382_MOESM1_ESM.pdf]

Epigenetic deregulation of GATA3 in neuroblastoma is associated with increased GATA3 protein expression and with poor outcomes

Bader Almutairi, Jessica Charlet, Anthony R. Dallosso, Marianna Szemes,

Heather C. Etchevers, Karim T. A. Malik, Keith W. Brown.

Supplementary data:

Supplementary figures S1 to S16

Supplementary tables S1 to S6

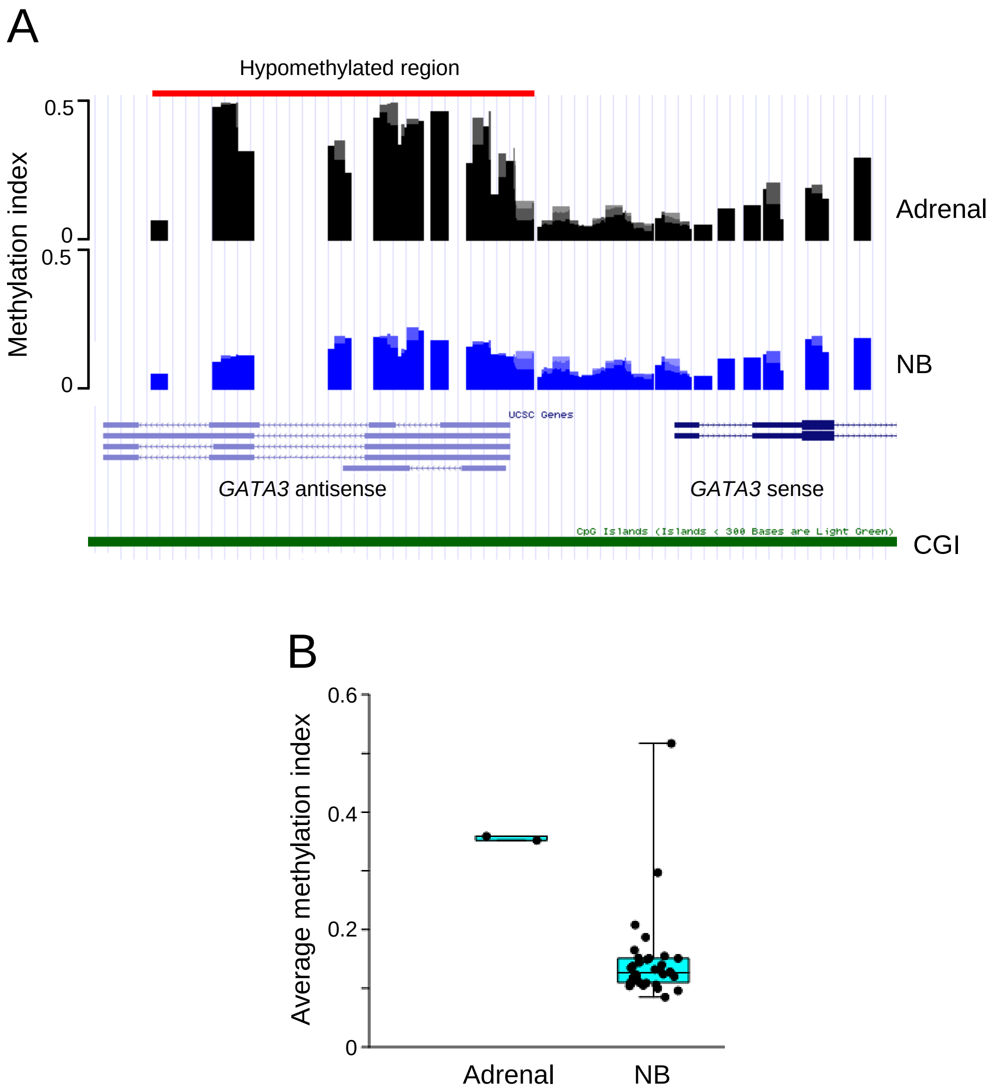

Figure S1: *GATA3* DNA methylation from public dataset GSE54719
A: DNA methylation index from Illumina Human Methylation 450K Beadchip analysis, showing the mean results from two adrenal gland samples (Adrenal) and the mean results from 35 neuroblastoma tumours (NB), across the *GATA3* locus, taken from GSE54719. Results were uploaded as a Bedgraph file to the UCSC genome browser (http://genome.ucsc.edu/), using GRCh37/hg19.
B: Boxdotplot of the methylation index of each sample, averaged across the hypomethylated region (shown in red at the top of part A).

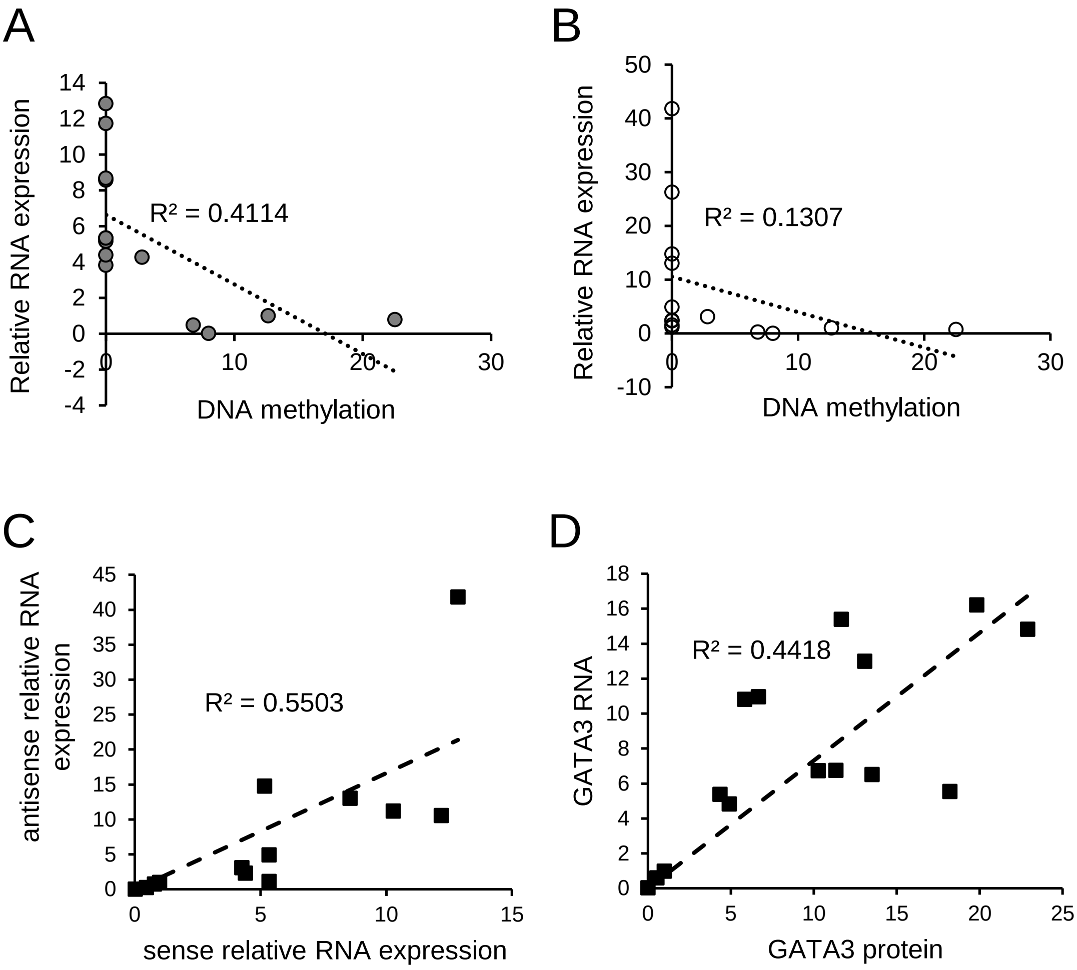

Figure S2: Correlations between *GATA3* DNA methylation, RNA and protein expression
GATA3 sense and antisense RNA expression, protein expression and DNA methylation in normal tissues (FA, hNCC, DRG/SG) and neuroblastoma cell lines. DNA methylation is the average of pyrosequencing assays 01 and 02. RNA levels were assayed by real-time PCR and normalised to the endogenous levels of *TBP*, and expressed relative to hNCC RNA. Protein levels were assayed by Western blotting relative to ACTIN, and expressed as a ratio of the level in FA.
A: *GATA3* sense RNA expression and DNA methylation.
B: *GATA3* antisense RNA expression and DNA methylation.
C: *GATA3* antisense and sense RNA expression.
D: *GATA3* sense RNA and protein expression.

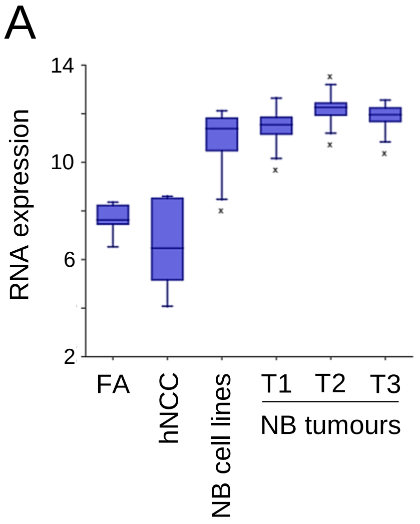

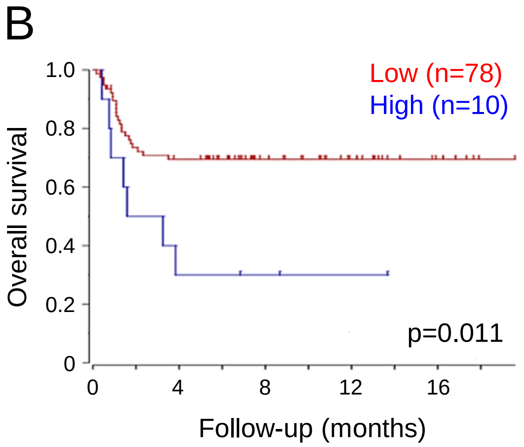

Figure S3: *GATA3* expression and survival data from R2

A: Boxplot of *GATA3* sense RNA expression determined by Affymetrix microarray, taken from R2 (https://hgserver1.amc.nl/cgi-bin/r2/main.cgi), using 13 samples of FA collected from different datasets (GSE3525, GSE7307, GSE8514), 5 samples of hNCC (GSE1430), 24 samples of NB cell lines (GSE28019) and three sets of NB tumours, containing 51 samples in T1 (GSE16237), 30 samples in T2 (GSE13136) and 88 samples in T3 (GSE16476). *GATA3* RNA expression was significantly upregulated in NB cell lines and three sets of NB tumours compared to normal tissues (FA and hNCC) (One Way ANOVA; $p = 6.4 \times 10^{-50}$).

B: Kaplan-Meier analysis demonstrates overall survival probability in NB tumour data set GSE16476. Tumours with a high level of *GATA3* RNA (“high”) (n=10) showed poorer outcomes compared to tumours expressing a low level of *GATA3* RNA (“low”) (n=78).

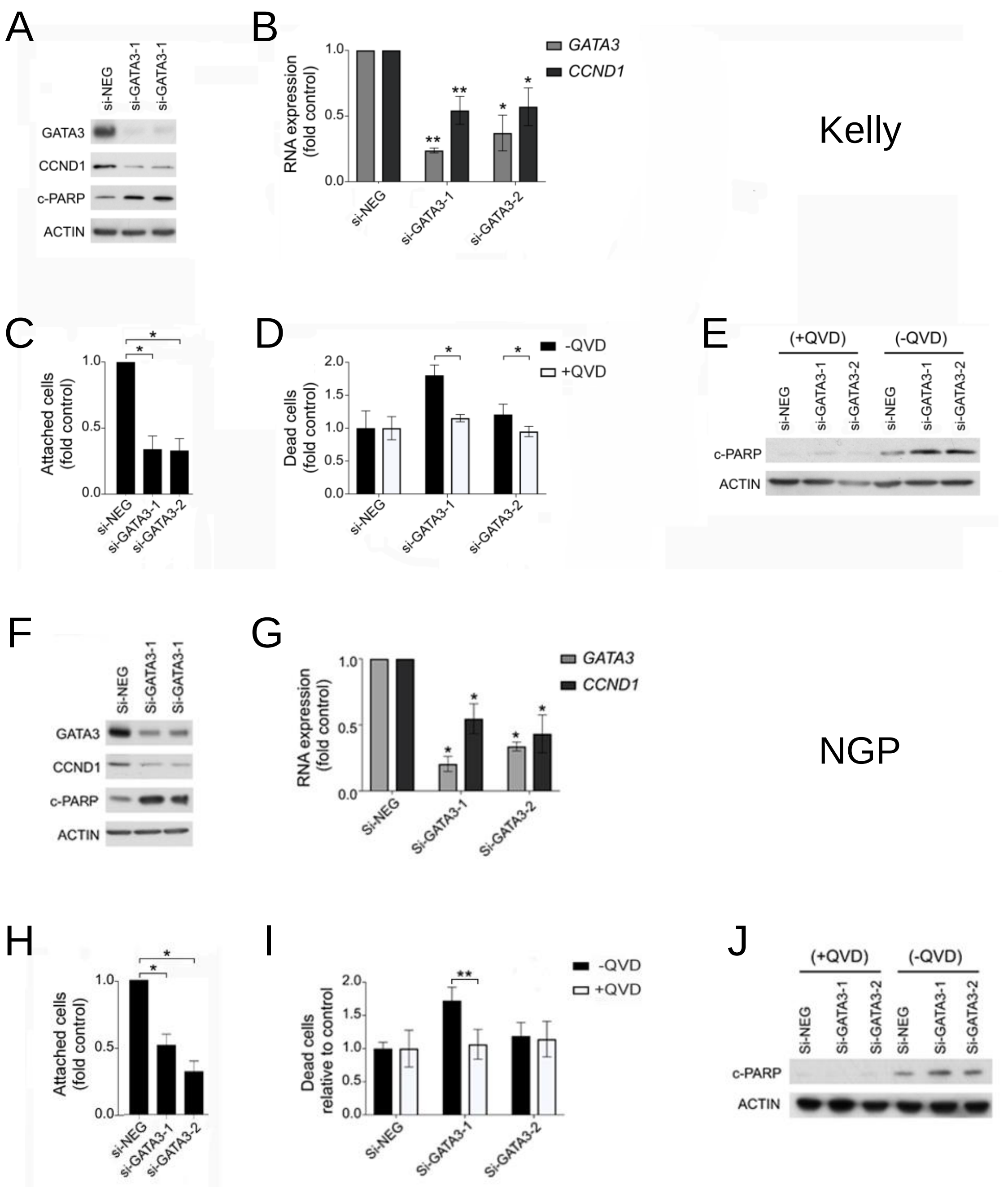

Figure S4: GATA3 biological functions

Growth of Kelly (A-E) or NGP (F-J) neuroblastoma cell lines after transfection with non-targeting siRNA (siNEG), or *GATA3* siRNAs (si-GATA3-1 and si-GATA3-2).

A, F: Western Blot of GATA3, cleaved PARP (c-PARP) and CCND1 protein expression, with ACTIN as a loading control.

B, G: GATA3 and CCND1 RNA expression assayed by QPCR expressed relative to siNEG.

C, H: Attached cell counts relative to siNEG controls.

D, I: Dead cell counts (floating cells that were trypan blue permeable) expressed relative to siNEG control in cells treated caspase 3 inhibitor QVD (+QVD, unfilled bars) or with drug solvent (DMSO; -QVD, filled bars).

E, J: Western Blot of c-PARP protein expression in transfected cells with (+QVD) or without (-QVD) QVD- treatment, with ACTIN as a loading control.

A, B, C, D, F, G, H, I; mean ± S.E.M, n=3; * $p<0.05$; ** $p<0.01$, t test. E, J; representative of n=3.

Uncropped blots are shown in supplementary figures S13 and S14.

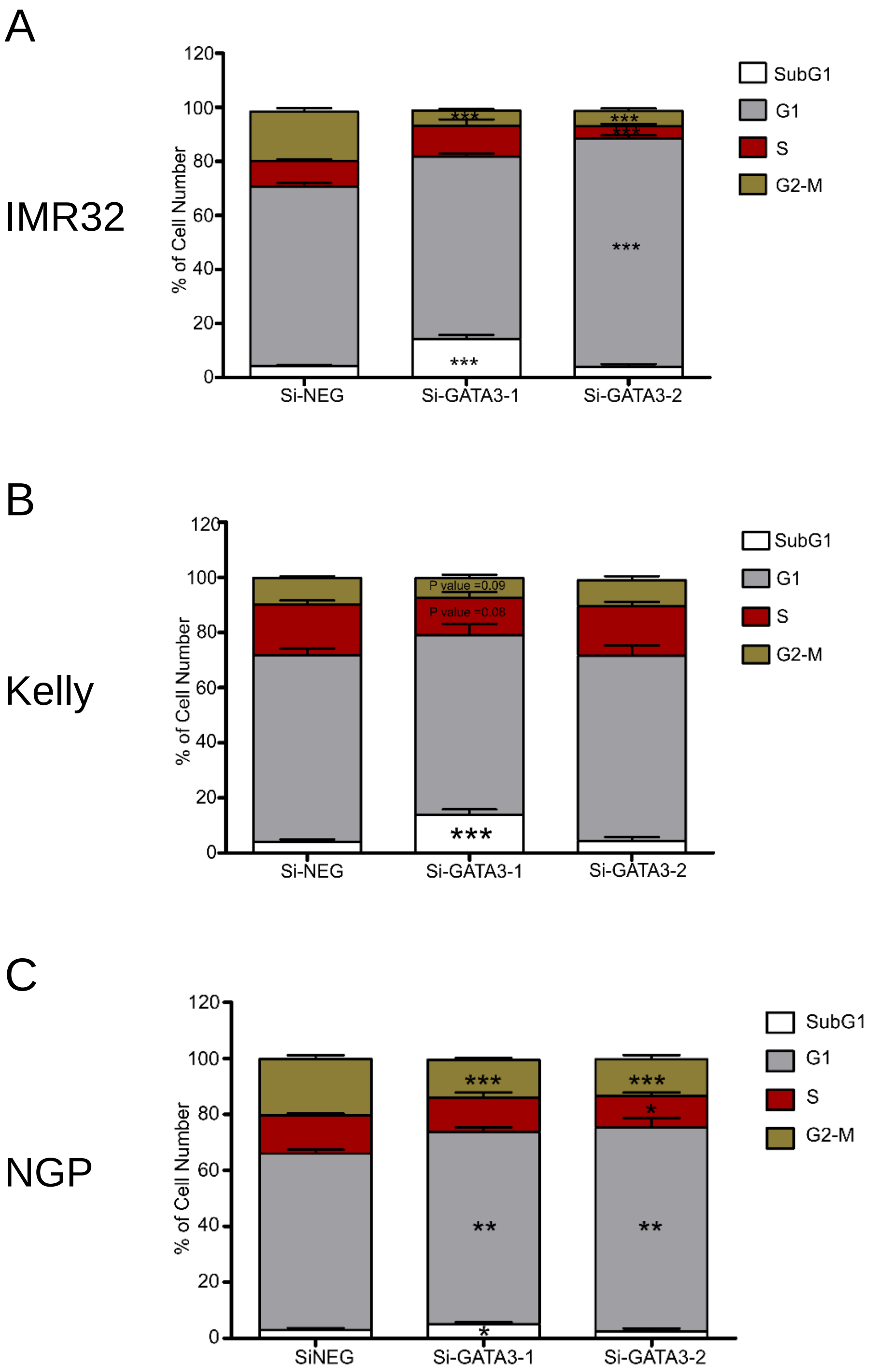

Figure S5: Effect of GATA3 depletion on cell cycle distribution
Cell cycle analysis by flow cytometry of cell lines 72hr after transfection with non-targeting siRNA (si-NEG) or with siRNAs targeting GATA3 (si-GATA3-1 and -2). Results shown are the mean ± standard deviation of three experiments.
A: IMR32, **B:** Kelly, **C:** NGP.
***p<0.001; unpaired t test; in comparison to si-NEG.

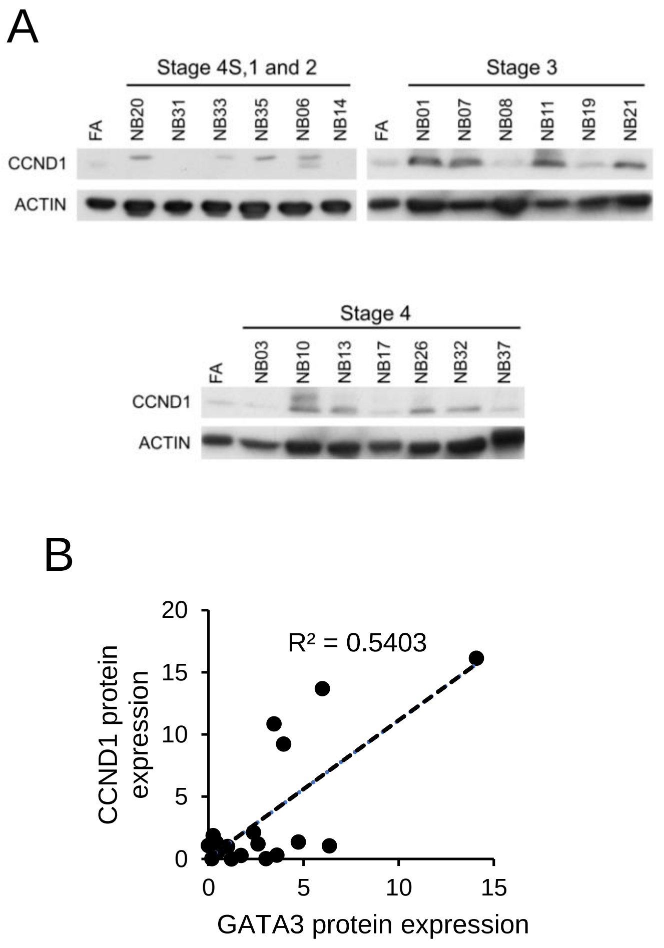

Figure S6: CCND1 expression in neuroblastoma

A: CCND1 protein levels assayed by Western blot in fetal adrenal (FA) and 19 neuroblastomas (NB) with ACTIN as loading control. Uncropped blots are shown in supplementary figure S15.

B: Correlation between GATA3 and CCND1 protein expression in neuroblastoma tumours (n=19).

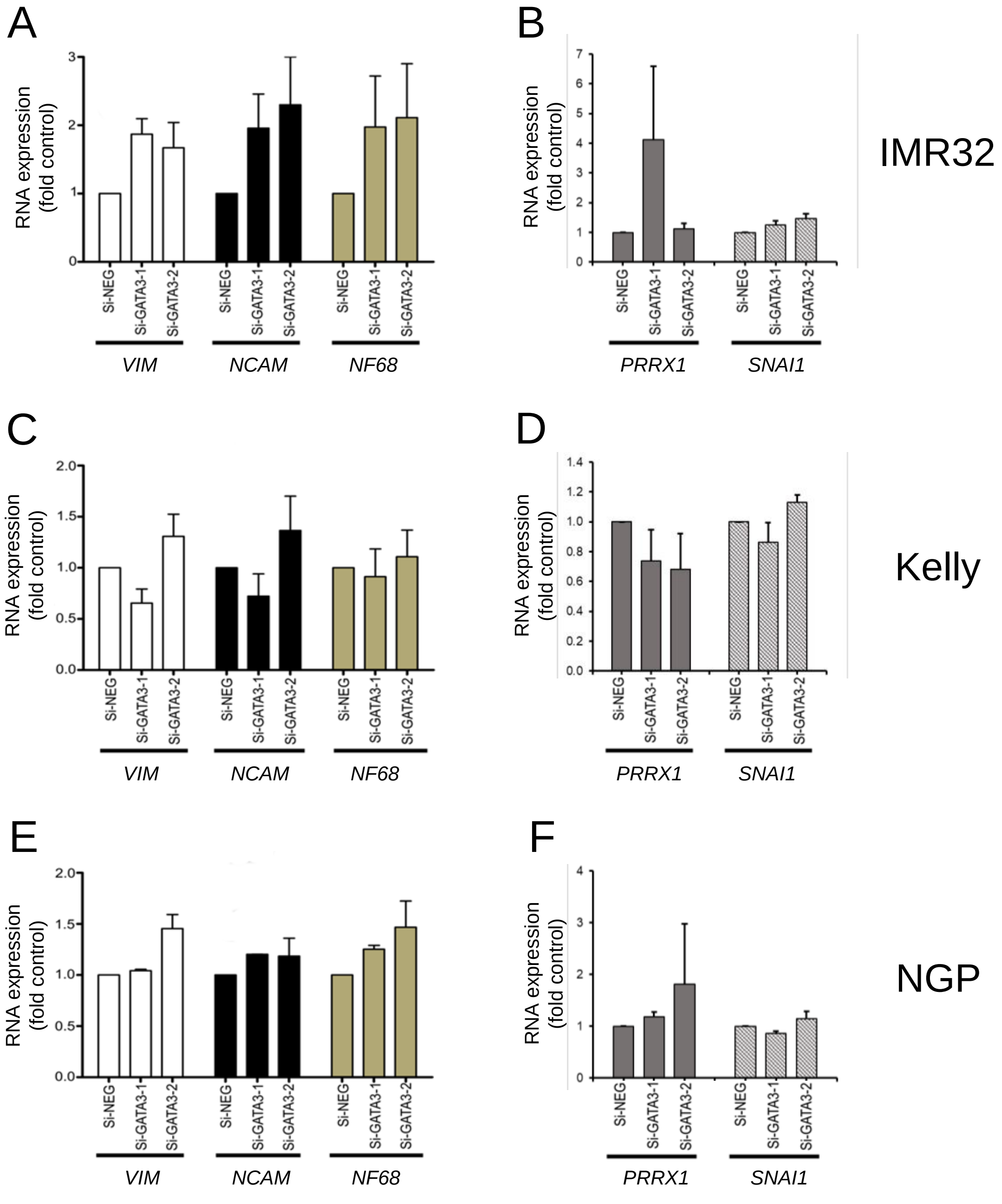

Figure S7: Differentiation markers in NB cell lines
RNA expression of *VIM*, *NCAM* and *NF68* (A, C, E), *PRRX1* and *SNAI1* (B, D, F) in IMR32 (A, B), Kelly (C, D) and NGP (E, F) cell lines, 72 hours after siGATA3 transfection. RNA expression was assayed by QPCR, normalised to the housekeeping gene *TBP* and expressed relative to control (siNEG). Results shown are mean ± S.E.M, n=3.

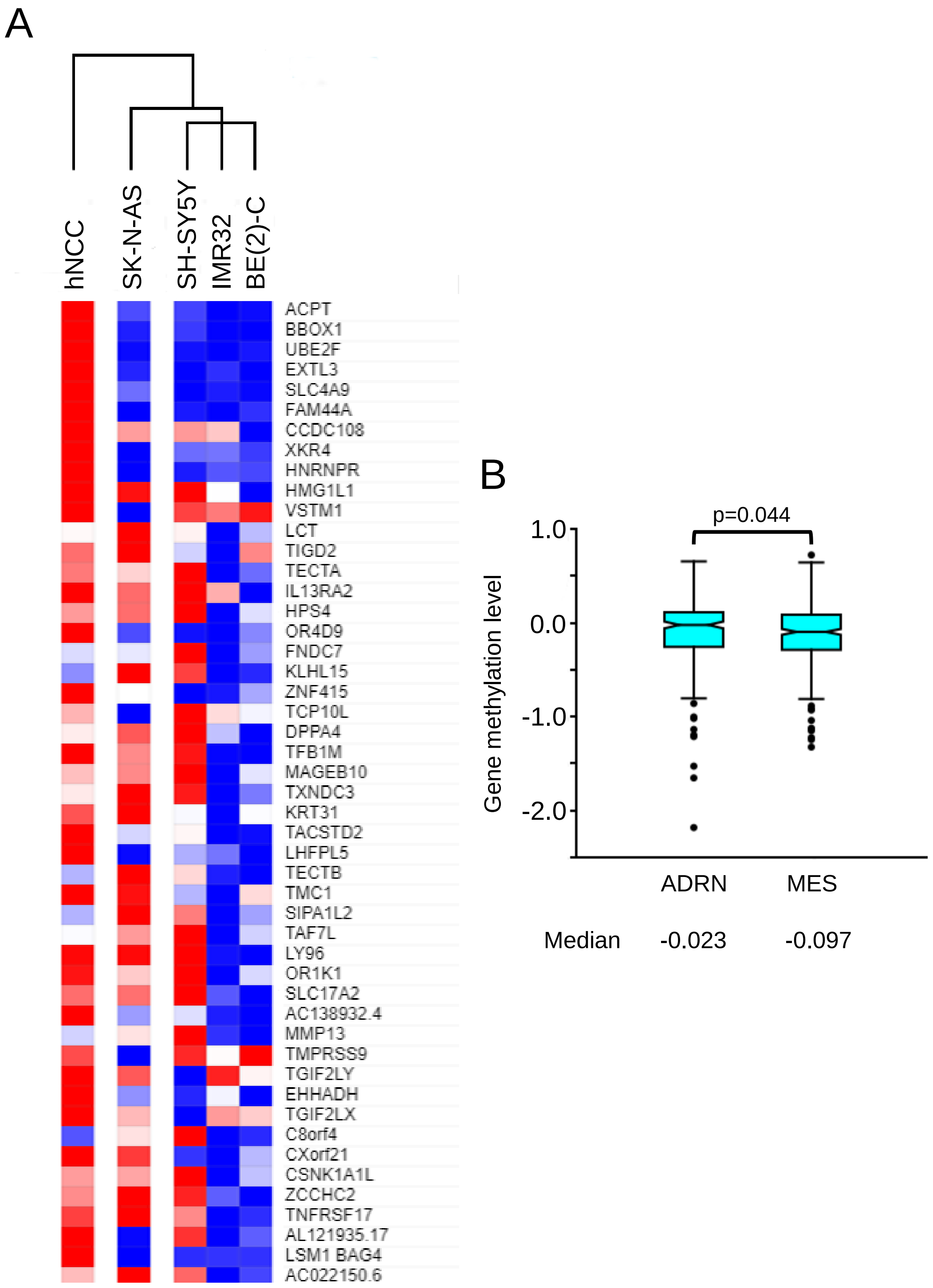

Figure S8: DNA methylation clustering of neural crest and neuroblastoma cell lines, and DNA methylation of ADRN and MES signature genes in neural crest cells

A: Hierarchical clustering of neural crest cells (hNCC) and four neuroblastoma cells lines by DNA methylation, using one minus Pearson clustering (https://software.broadinstitute.org/morpheus/). Only a portion of the heat map is shown below the hierarchical cluster.

B: Boxplot of DNA methylation levels in hNCC of adrenergic (ADRN) and mesenchymal (MES) signature genes (gene lists from van Groningen et al (2017) Nat Genet 49:1261-1266). p value from Mann-Whitney test.

Gene methylation levels were derived from the mean probe ratios of the differentially methylated probes within 700bp of the transcriptional start site (log2), from our previously published MCIP study (Charlet et al (2017) Mol Carcinogenesis 56: 1290-1301).

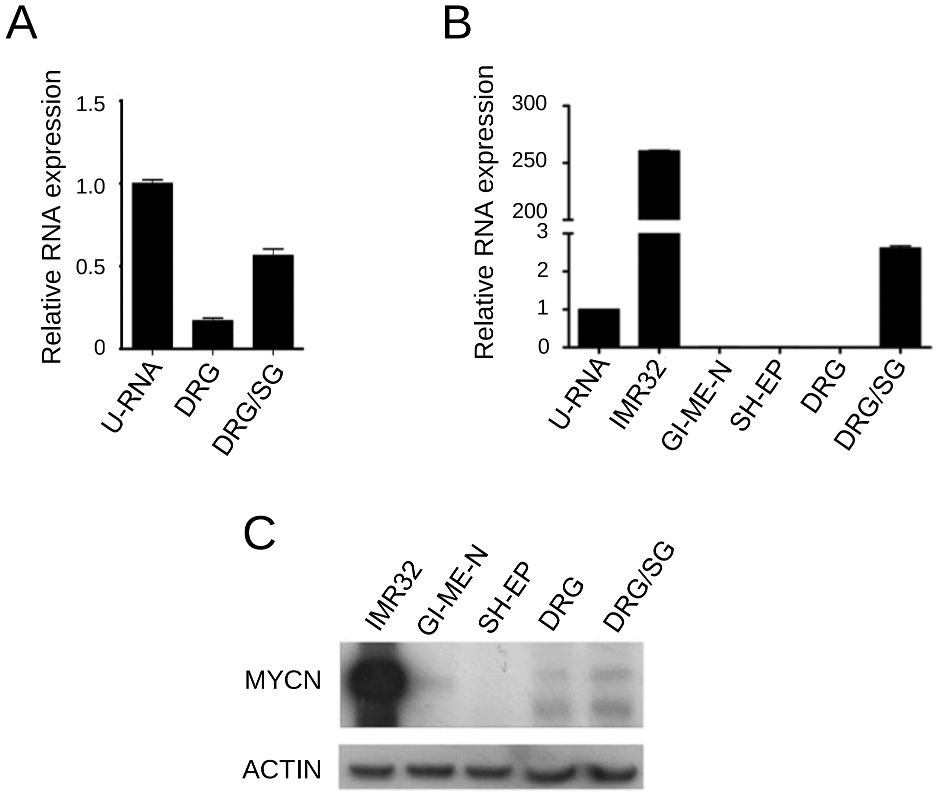

Figure S9: DRG/SG express *DBH* and *MYCN*
A: *DBH* RNA expression in universal RNA (U-RNA), dorsal root ganglia cultured cells (DRG) and dorsal root ganglia/sympathetic ganglia cultured cells (DRG/SG).
B: *MYCN* RNA expression in U-RNA, IMR32 (amplified *MYCN*), GI-ME-N (unamplified *MYCN*), SH-EP (unamplified *MYCN*), DRG and DRG/SG.
In A and B, RNA levels were assayed by real-time PCR and normalised to the endogenous levels of *TBP*, and expressed relative to U-RNA.
C: Western blot of MYCN protein expression in IMR32 (amplified *MYCN*), GI-ME-N (unamplified *MYCN*), SH-EP (unamplified *MYCN*), DRG and DRG/SG, with ACTIN as the loading control. Uncropped blots are shown in supplementary figure S16.

Figure 3B

GATA3

ACTIN

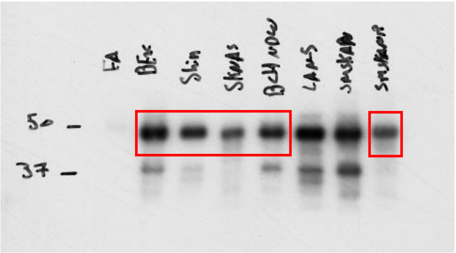

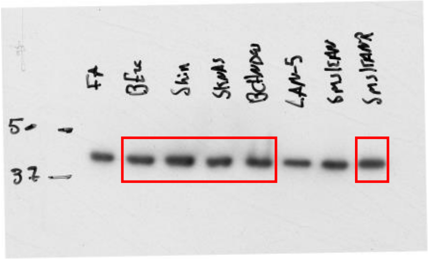

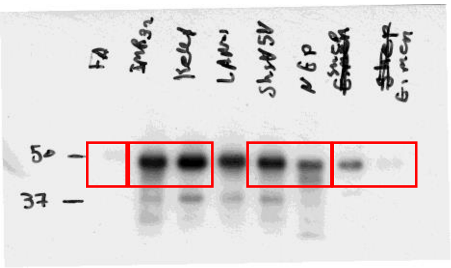

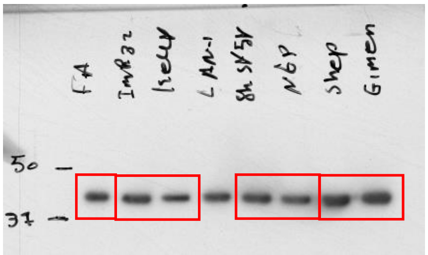

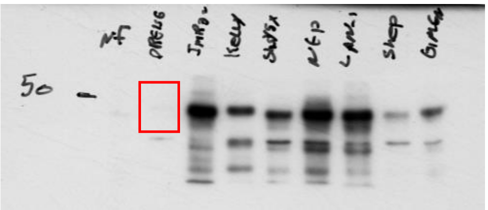

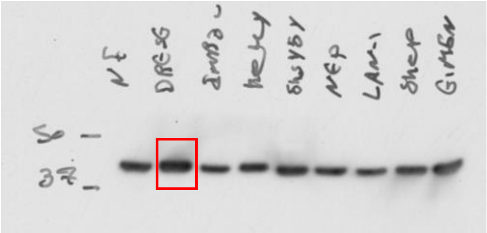

Figure 3C

GATA3

ACTIN

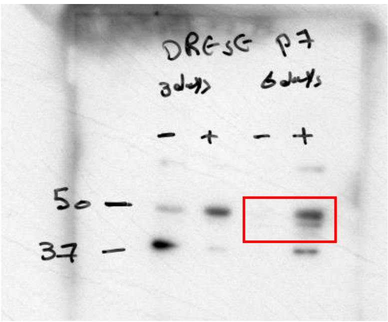

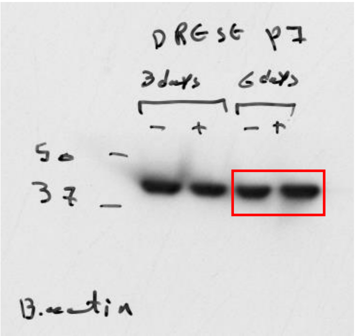

Figure S10: Uncropped blots for figure 3
Areas used in the figure are outlined by red boxes

Figure 4C

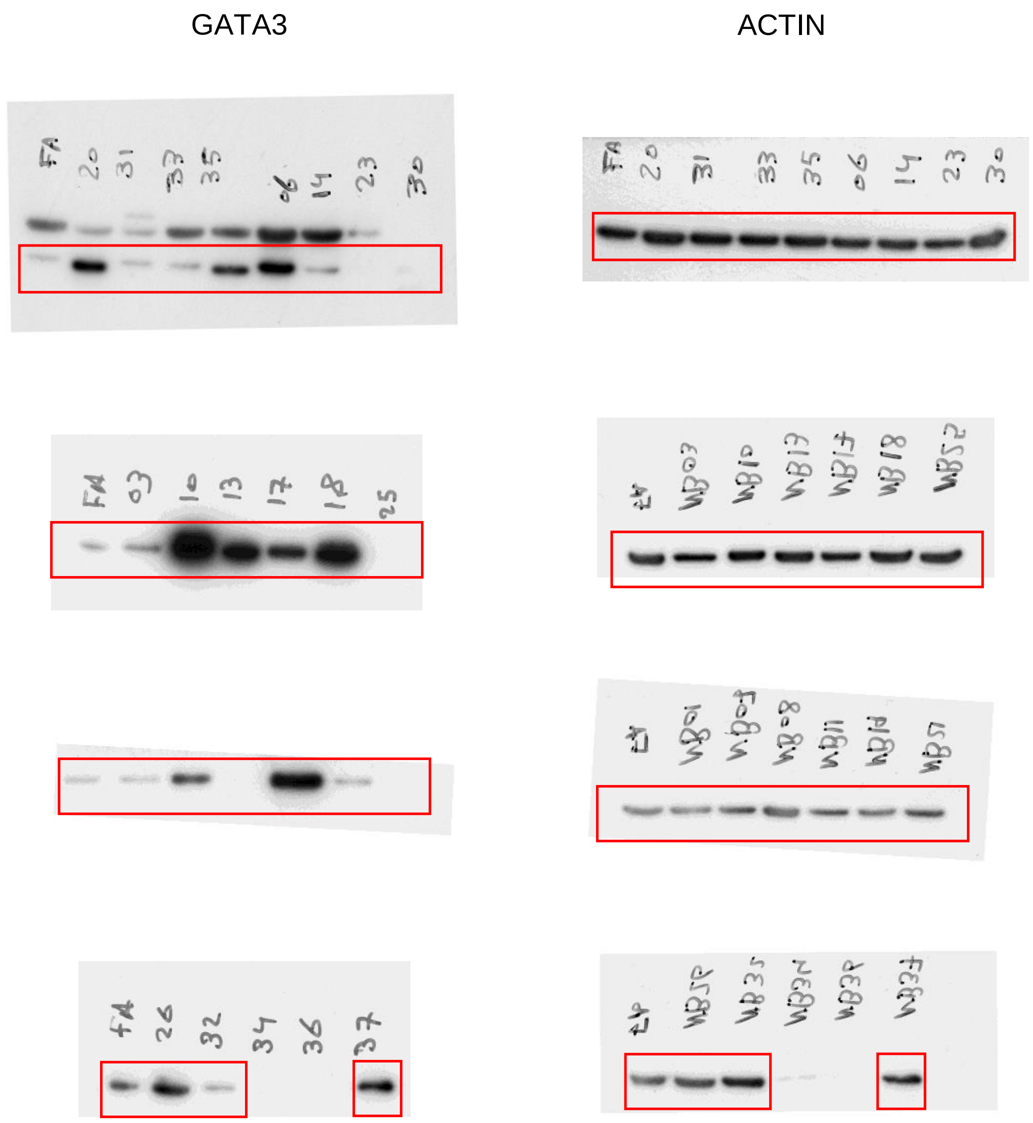

Figure S11: Uncropped blots for figure 4
Areas used in the figure are outlined by red boxes

Figure 5A

GATA3

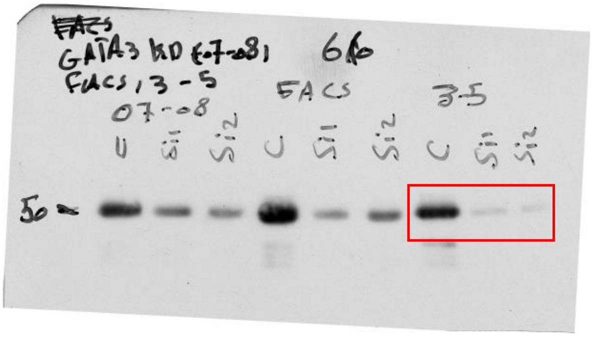

CCND1

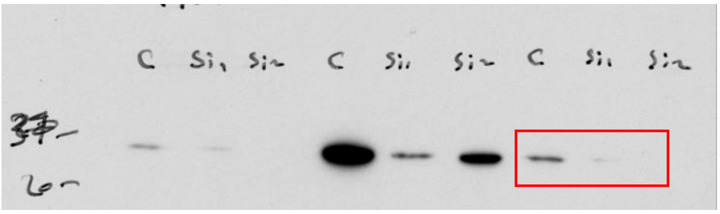

C-PARP

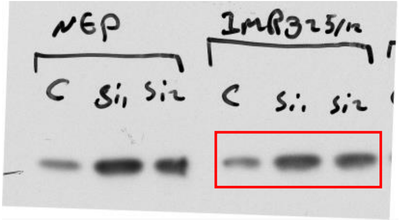

ACTIN

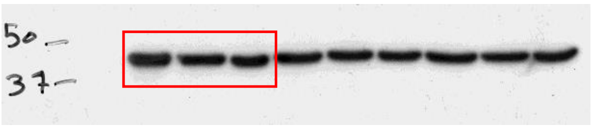

Figure 5E

C-PARP

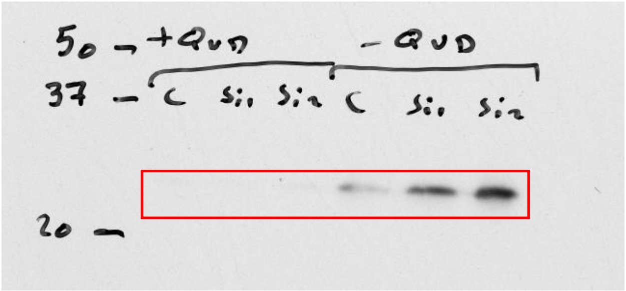

ACTIN

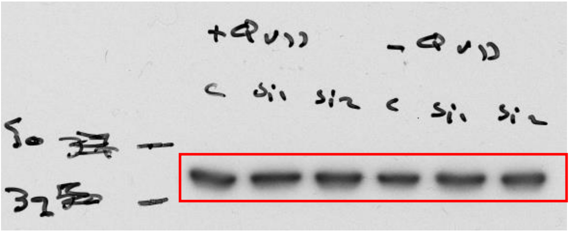

Figure S12: Uncropped blots for figure 5
Areas used in the figure are outlined by red boxes

Figure S4A

GATA3

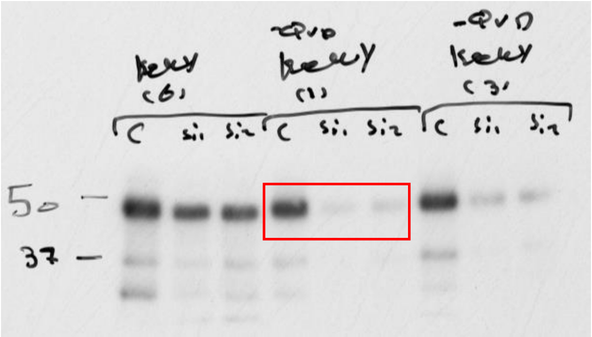

CCND1

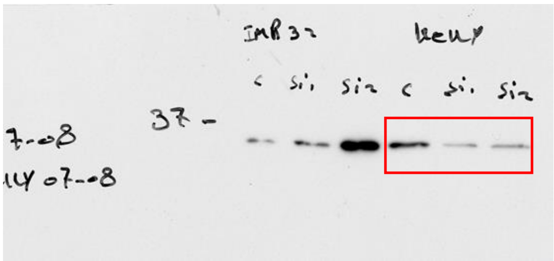

C-PARP

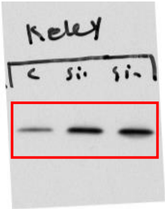

ACTIN

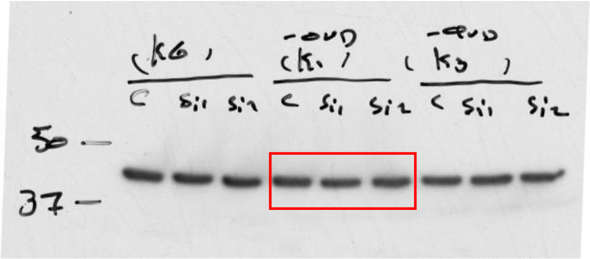

Figure S4E

C-PARP

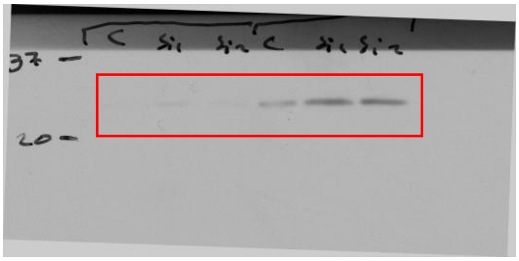

ACTIN

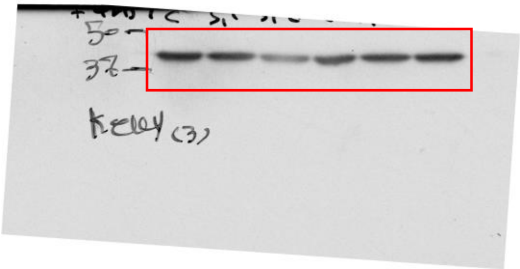

Figure S13: Uncropped blots for supplementary figure S4A, E
Areas used in the figure are outlined by red boxes

Figure S4F

GATA3

CCND1

C-PARP

ACTIN

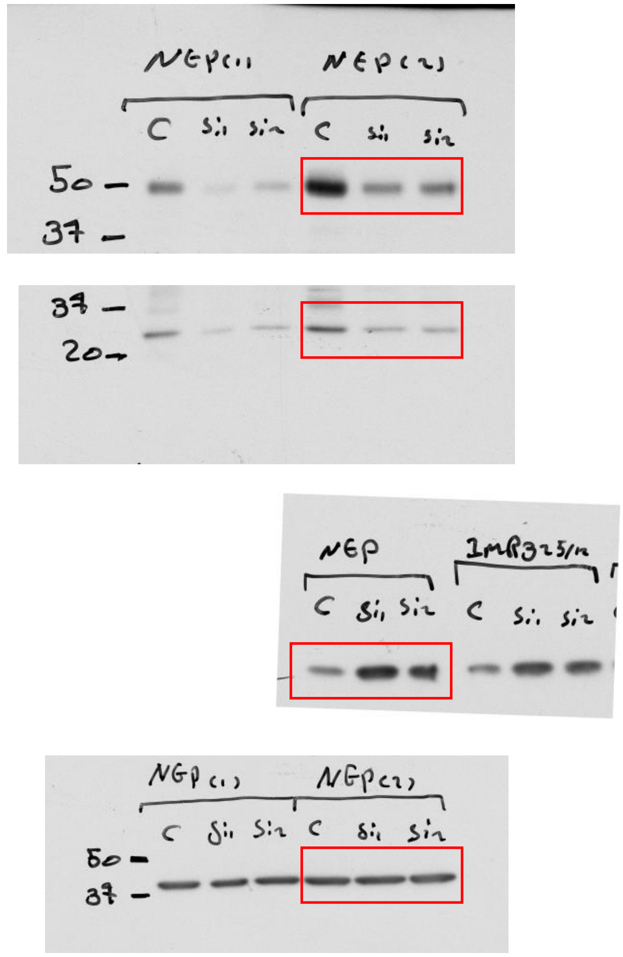

Figure S4J

C-PARP

ACTIN

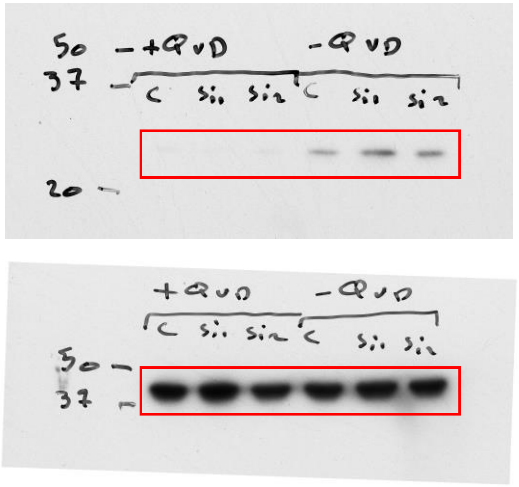

Figure S14: Uncropped blots for figure S4F, J
Areas used in the figure are outlined by red boxes

Figure S6A

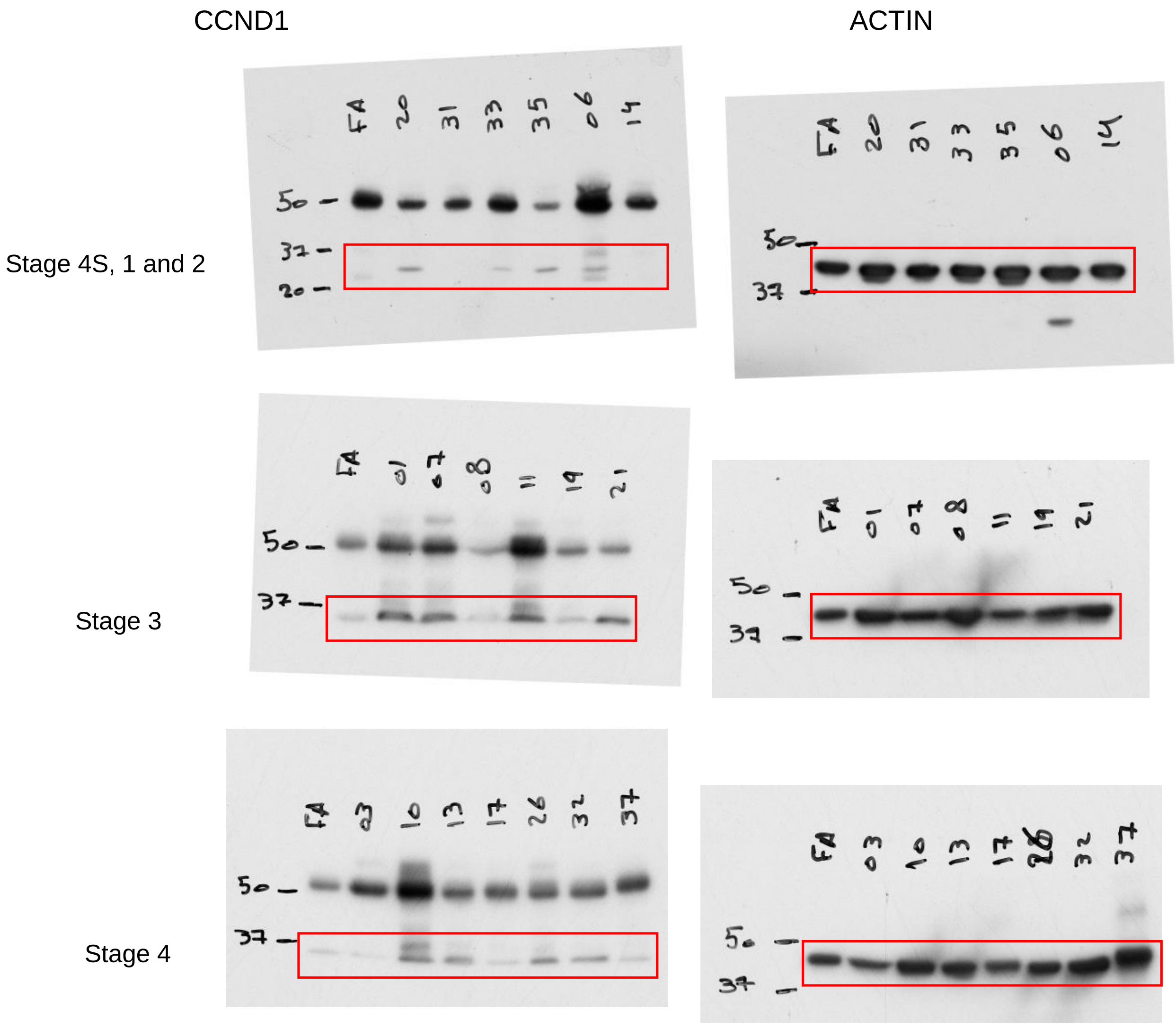

Figure S15: Uncropped blots for figure S6A
Areas used in the figure are outlined by red boxes

Figure S9C

MYCN

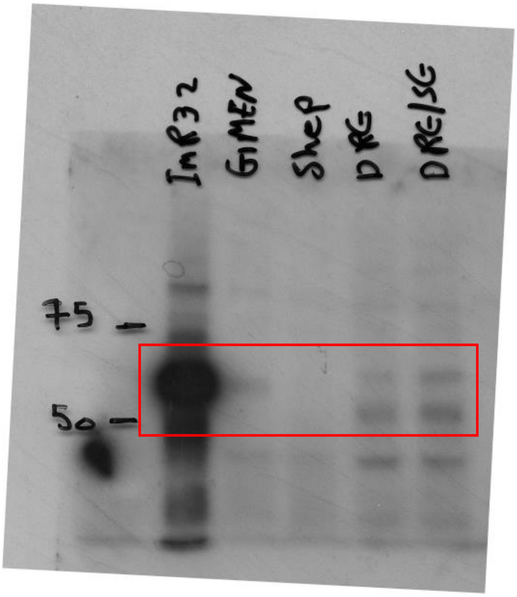

ACTIN

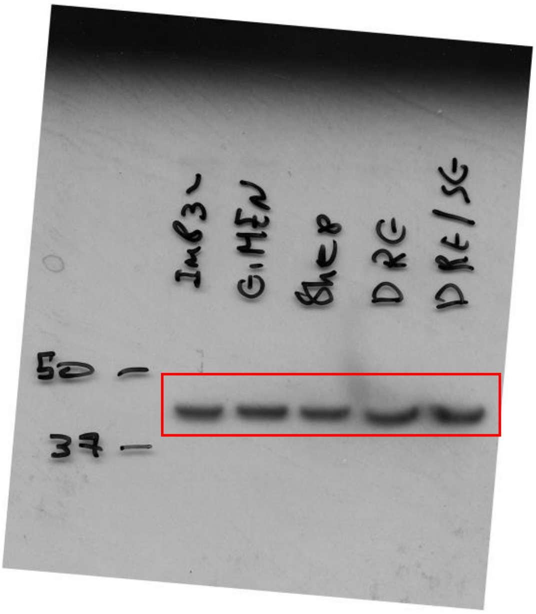

Figure S16: Uncropped blots for supplementary figure S9
Areas used in the figure are outlined by red boxes

Table S1: Hypomethylated genes - survival and expression data

Analysed in R2 (http://r2.amc.nl) using neural crest (hNCC) - Etchevers - GSE14340, neuroblastoma tumours - Versteeg - GSE16476, neuroblastoma cells lines - Versteeg - GSE28019 for expression analysis and Versteeg GSE16476 for survival analysis.

"Y" in NB survival indicates poorer relapse-free survival patients with low gene expression and $p<0.05$.

"Y" in NB expression indicates reduced expression in both NB cell lines and NB tumours compared to hNCC with $p<0.05$.

Gene methylation values and CGI data from table S6 in Charlet, J., et al (2017) Mol. Carcinog. 56(4): 1290-1301.

Gene methylation levels were derived from the mean probe ratios of the differentially methylated probes within 700bp of the transcriptional start site (log2).
PRC marked indicates those genes marked by PRC2 in ES cells.
LCF, ICF and HCF indicate low-, intermediate- or high-CpG promoters.

	Gene methylation values (log_2)									p<0.05 only		R2 overall survival	GATA3 RNA expression compared to hNCC			
										High=poor	NB>hNCC	low poor / high poor		UP in NB vs hNCC / DOWN in NB vs hNCC		
Genes	hNCC	SK-N-AS	SH-SY5Y	IMR32	BE(2)-C	PRC marked	LCP	ICP	HCP	NB survival	NB expression	P	hNCC median	NB cell line median	NB tumour median	Anova P
ABCA7	-0.042	-1.232	-1.243	-0.956	-0.949			Y				4.90E-02	5.31	4.87	5.16	5.80E-03
AR	0.370	-0.571	-0.849	-0.509	-0.453			Y				2.50E-02	7.17	3.9	4.01	2.40E-08
ATP13A5	0.221	-1.361	-1.210	-0.416	-1.344		Y					4.60E-02	3.42	3.06	3.38	7.90E-01
CCL3	0.237	-1.143	-1.770	-1.184	-1.009		Y					1.12E-01	1.26	1.2	6.74	5.50E-42
CD69	0.349	-1.259	-0.931	-0.770	-0.825		Y				Y	8.50E-04	0.00	0	5.66	6.70E-29
CDYL	0.368	-1.052	-0.743	-0.882	-0.672					Y		3.10E-04	7.52	7.83	6.34	1.10E-03
CLEC4D	0.277	-1.293	-1.217	-0.862	-0.774		Y				Y	9.50E-02	3.04	3.32	3.78	4.00E-02
CTSG	0.288	-0.961	-1.005	-0.997	-0.811		Y			Y		2.20E-03	3.09	2.60	4.08	7.00E-06
CYP2A7	-0.690	-2.392	-2.105	-1.889	-1.989	Y	Y					4.90E-02	3.62	2.62	3.83	2.00E-03
DARC	0.148	-2.054	-1.467	-1.328	-0.793		Y			Y	Y	4.20E-02	1.14	1.54	5.31	2.10E-15
DDR2	0.288	-1.591	-1.604	-1.009	-1.282		Y			Y	Y	5.60E-07	9.01	9.41	7.58	5.80E-16
DEFA1	-0.148	-1.607	-1.926	-1.415	-1.128		Y				Y	3.30E-02	1.54	3.69	5.63	1.40E-11
DEFB104B	0.271	-1.821	-1.792	-0.868	-1.491		Y			Y		2.40E-02	3.26	2.64	2.70	2.90E-01
DEFB128	0.070	-1.459	-1.231	-1.269	-1.119		Y									
DLC1	0.456	-0.650	-0.404	-0.320	-0.597	Y	Y					2.70E-11	11.83	7.11	9.58	4.80E-16
DPCR1	-0.657	-3.584	-3.675	-3.113	-3.079		Y			Y		1.40E-03	2.51	2.56	2.15	7.00E-01
EYA1	0.339	-0.725	-0.742	-0.629	-0.747			Y			Y	2.70E-02	0.49	7.85	8.63	2.60E-12
FAM103A1	0.281	-0.499	-0.800	-0.414	-0.510				Y			2.50E-02	9.00	8.74	8.58	1.80E-01
FCGR2B	0.076	-1.860	-1.618	-1.348	-0.971		Y			Y		1.90E-03				
FRMD4A	0.460	-0.443	-0.624	-0.411	-0.713		Y					2.30E-04				
GATA3	-0.090	-1.868	-1.772	-1.343	-1.554	Y			Y	Y	Y	1.10E-02	6.47	11.39	11.55	1.90E-19
GRIK2	0.290	-1.047	-0.983	-0.334	-1.002				Y		Y	1.20E-02	1.14	2.04	4.95	1.00E-14
GZMA	0.347	-0.787	-0.879	-0.500	-1.054		Y					1.70E-01	3.05	2.16	5.41	1.10E-19
HBG1	0.320	-1.561	-1.379	-1.054	-0.832		Y					1.70E-03	2.04	1.72	7.60	2.10E-19
HBG2	0.302	-1.304	-0.970	-0.893	-0.666							1.70E-03	2.04	1.72	7.60	2.10E-19
HLA-DPA1	0.376	-1.575	-1.548	-0.977	-1.066		Y					8.60E-02	7.05	2.82	10.82	1.40E-41
HTN1	0.594	-1.019	-0.789	-0.604	-0.659		Y			Y		2.90E-02	3.26	2.72	2.94	7.20E-01
HTN3	0.530	-0.930	-0.899	-0.570	-0.960			Y		Y	Y	1.00E-02	0.00	0.00	1.98	1.00E-02
IGFL2	0.176	-1.943	-0.951	-1.434	-1.069		Y			Y		4.60E-02	1.77	1.46	1.38	7.20E-01
IGFL4	0.155	-1.577	-2.044	-1.363	-1.743		Y									
KIR3DL3	0.393	-0.952	-1.388	-0.423	-0.789		Y			Y		1.40E-03	0.58	0.68	1.07	3.00E-01
KRTAP4-5	0.489	-0.070	-1.334	-0.940	-1.291		Y					7.00E-02	4.09	3.20	3.41	5.80E-01
LCE1A	-0.166	-1.380	-1.456	-1.338	-1.194		Y									
LILRA3	0.054	-1.185	-1.120	-0.790	-0.868		Y			Y		2.30E-02	0.93	0.54	1.00	6.60E-03
LILRB2	0.315	-1.953	-1.749	-0.975	-1.431		Y			Y		9.50E-03	1.07	0.72	4.37	1.60E-16
MED12L	0.358	-0.521	-0.503	-0.286	-0.422						Y	2.66E-01	2.07	3.72	3.80	4.00E-02
MGP	0.577	-0.925	-1.173	-0.085	-0.754		Y			Y	Y	8.90E-03	1.32	2.08	10.02	8.40E-28
MYH13	0.562	-0.733	-0.680	-0.652	-0.462		Y					1.45E-01	3.09	2.36	2.51	2.10E-01
MYT1	0.553	-0.626	-1.562	-0.994	-1.357						Y	1.90E-03	1.81	6.75	8.32	2.50E-26
NBEA	0.485	-1.123	-1.278	-1.128	-1.256			Y			Y	1.10E-02	6.71	9.34	9.92	1.70E-17
NLRP11	0.679	-1.287	-1.022	-0.605	0.104		Y					4.00E-02	0.85	2.93	2.17	1.80E-01
OR10G2	0.353	-1.662	-1.943	-1.361	-1.371		Y									
OR10K2	-0.424	-2.153	-1.617	-1.720	-1.492		Y									
OR14I1	0.113	-1.299	-0.835	-0.799	-0.884		Y									
OR1B1	0.270	-2.663	-1.953	-1.363	-1.681		Y									
OR1D4	0.218	-1.298	-1.565	-1.223	-1.262		Y									
OR1G1	-0.393	-1.243	-1.747	-1.475	-1.119		Y					2.51E-01	3.02	1.61	2.00	4.90E-01
OR1N1	0.240	-0.834	-0.545	-0.388	-0.959		Y									
OR2C3	-0.108	-1.468	-1.254	-0.952	-0.911		Y				Y	3.50E-02	0.00	0.53	2.25	6.20E-03
OR2D2	0.364	-1.722	-1.595	-1.393	-1.181		Y									
OR2T29	0.078	-1.536	-1.098	-1.238	-0.923		Y									
OR2T3	-0.047	-1.740	-1.244	-1.143	-0.987		Y									
OR2T34	-0.036	-1.772	-1.146	-1.241	-0.935		Y									
OR2T5	0.254	-1.721	-1.360	-1.294	-1.125		Y									
OR3A4	0.683	-0.900	-1.411	-0.599	-0.324		Y									
OR4D1	0.009	-1.083	-0.948	-1.127	-0.758		Y					5.60E-02	3.35	3.18	3.69	2.00E-02
OR6C70	0.095	-0.799	-0.688	-0.657	-0.664		Y									
OR6K6	0.387	-1.394	-0.987	-0.991	-0.554		Y									
OR8D1	0.034	-0.744	-1.229	-0.718	-1.040		Y			Y		4.90E-02	3.74	3.69	3.85	1.30E-01
PATE1	-0.035	-1.204	-1.801	-1.032	-1.750		Y			Y	Y	3.20E-02	0.38	2.87	2.22	9.10E-03
PRB1	-0.251	-1.516	-1.372	-0.869	-1.228		Y					1.55E-01	3.83	4.03	4.02	3.70E-01
RLN2	0.161	-0.401	-0.865	-0.451	-0.665			Y		Y		1.40E-03	3.96	4.26	4.56	2.10E-01
SPINLW1	-0.314	-1.520	-1.401	-1.084	-1.251		Y				Y	9.70E-02	0.00	2.37	3.46	4.90E-07
SPRYD5	-0.053	-1.155	-1.110	-0.876	-1.086		Y					4.90E-02	2.66	1.48	1.35	1.00E-01
TP53TG3	0.708	-0.338	-0.625	-0.012	-0.497			Y			Y	3.91E-01	3.60	6.11	7.28	7.60E-12
UGT2B11	-0.190	-2.051	-1.675	-1.562	-1.684		Y					2.20E-02	0.00	0.00	0.68	1.70E-01
UGT2B28	-0.058	-1.916	-1.492	-1.352	-1.643		Y					2.80E-02	6.55	1.07	3.10	3.10E-06

Table S2: Gene ontology of hypomethylated genes

Gene ontology of the genes identified by Chipmonk software as being hypomethylated between neural crest and neuroblastoma cell lines - see Table S5 for full list. Gene ontology was assessed using the statistical overrepresentation test in PANTHER (http://www.pantherdb.org/). Only results with P<0.05 are shown.

	Reference list	Client Text Box Input
Mapped IDs:	16906 out of 16844	65 out of 70
Unmapped IDs:	7125	2
Multiple mapping information:	1509	5

	2006-11-02_HG18_CpG_Promo gene list.txt (REF)	Client Text Box Input					
**PANTHER GO-Slim Biological Process**	**#**	**#**	**expected**	**Fold Enrichment**	**+/-**	**raw P value**	**FDR**
**regulation of biological process**	2143	23	8.9	2.6	+	0.0000112	0.000303
biological regulation	2637	26	11.0	2.4	+	0.0000168	0.00041
response to stimulus	2337	31	9.7	3.2	+	7.54E-10	6.13E-08
**G-protein coupled receptor signaling pathway**	404	11	1.7	6.6	+	0.000001	0.0000489
cell surface receptor signaling pathway	1033	16	4.3	3.7	+	0.00000442	0.000154
**sensory perception of smell**	210	12	0.9	13.8	+	1.06E-10	2.58E-08
sensory perception of chemical stimulus	248	12	1.0	11.6	+	6.52E-10	7.95E-08
sensory perception	387	13	1.6	8.1	+	8.34E-09	0.000000509
neurological system process	856	15	3.6	4.2	+	2.13E-06	0.0000865
system process	945	15	3.9	3.8	+	6.98E-06	0.000213
single-multicellular organism process	1474	18	6.1	2.9	+	2.56E-05	0.000568
multicellular organismal process	1488	18	6.2	2.9	+	2.90E-05	0.00059

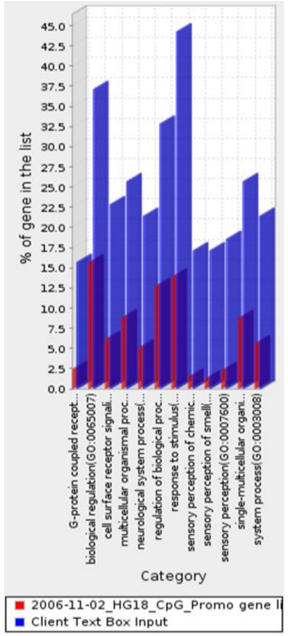

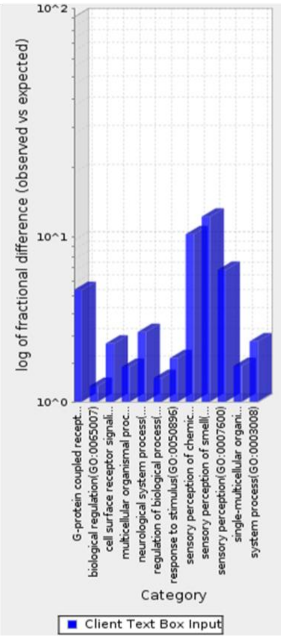

Table S3: Neuroblastoma cell lines used in the study

N = neuronal subtype, S = substrate adherent subtype, I = intermediate subtype
M = male, F = female
LN = lymph node, BM = bone marrow
Blank = not known.

Data taken from:

Thiele, C. J. (1998) Neuroblastoma cell lines, Journal of Human Cell Culture 1: 21-53
Reynolds CP, et al (1986) Human Neuroblastoma Cell-Lines Established before and after Therapy. Journal of the National Cancer Institute, 76(3):375-87
Cornaglia-Ferraris P, et al (1990) A new human highly tumorigenic neuroblastoma cell line with undetectable expression of N-myc. Pediatr Res, 27(1):1-6
BCH-N-DW: personal communication, C. McConville

Cell line	Cell type	Parental cell line	Patient age (months)	Sex	Primary site	Metastatic site	Cell line origin	Treated	MYCN amplification	Stage
**BE(2)-C**	I	SK-N-BE(2)	26	M		BM	BM	Yes	Yes	4
**BE(2)-M17**	N									
**BCH-N-DW**	N & S	-	17	M	Abdomen	BM	BM	No	Yes	4
**GI-ME-N**	S	-	24	F	Adrenal	LN, BM	BM	Yes	No	4
**IMR32**	N	-	13	M	Abdomen		Abdomen	No	Yes	
**Kelly**	N	-	12	F					Yes	
**LAN-1**	N	-	24	M		BM	BM		Yes	4
**LAN-5**	N	-	5	M		BM	BM		Yes	4
**NGP**	N	-	30	M		Lung	Lung		Yes	4
**SH-EP**	S	SK-N-SH	48	F	Thorax	BM	BM	Yes	No	4
**SH-IN**	I									
**SH-SY5Y**	N									
**SK-N-AS**	I	-	96	F	Adrenal	BM	BM	Yes	No	4
**SMSKAN**	N	-	36	F	Pelvic	BM	Pelvis	No	Yes	4
**SMSKANR**	N	-	44	F	Pelvic	BM	BM	Yes	Yes	4

Table S4: Neuroblastoma tumours used in the study

Blank = not known, ND = not done.

Lab code	Diagnosis	Stage	Relapse	Death	MYCN amplification
**NB01**	NB	3	No	No	No
**NB03**	NB	4	Yes	Yes	ND
**NB06**	NB	2	Yes	No	No
**NB07**	NB	3	Yes	Yes	Yes
**NB08**	Ganglio NB	3	No	No	No
**NB10**	NB	4	Yes	Yes	Yes
**NB11**	NB	3	Yes	Yes	No
**NB13**	NB	4	No	No	Yes
**NB14**	Ganglio NB	2	Yes	No	No
**NB16**	NB in situ	------	-----	Yes[1]	No
**NB17**	NB	4	Yes	Yes	No
**NB18**	NB	4	Yes	Yes	Yes
**NB19**	NB	3	No	No	No
**NB20**	NB	4S	Yes	No	No
**NB21**	NB	3	No	No	No
**NB23**	Ganglio NB	2	No	No	No
**NB25**	NB	4	Yes	Yes	No
**NB26**	NB	4	Yes	Yes	No
**NB27**	Ganglio NB		No	No	No
**NB30**	NB	2	No	No	No
**NB31**	NB	4S	No	No	No
**NB32**	NB	4	Yes	Yes	No
**NB33**	Ganglio NB	1	Yes	No	No
**NB35**	NB	1	No	No	ND
**NB37**	NB	4	No	No	ND

[1]Sudden infant death syndrome

Table S5: siRNAs used in the study

siRNA sequences taken from: Shan L, et al (2014) GATA3 cooperates with PARP1 to regulate CCND1 transcription through modulating histone H1 incorporation. Oncogene, 33(24):3205-16

siRNA	Sequence 5'-3'
si-GATA3-NEG	UUCUCCGAACGUGUCACGU
si-GATA3-1	CUCUGGAGGAGGAAUGCCA
si-GATA3-2	CUACAAGCUUCACAAUAUU

Table S6: Oligonucleotide primers used in the study

Application	Gene		5' - 3' sequence
QPCR	*CCND1*	F	ACTACCGCCTCACACGCTTC
		R	TTCGATCTGCTCCTGGCAG
	DBH	F	CCAATATCCCCGAACCGGAG
		R	GAAGCCCTTTGGAAGCTCCT
	GATA3 sense	F	TACGTGCCCGAGTACAGCTC
		R	ACAGTTCACACACTCCCTGC
	GATA3 antisense	F	TAGAAGAACCGTCCCTGAA
		R	CTTAGCAAAAATGCGTGTGC
	MYCN	F	TTTTAGATCTATTAAAACGAACGGGGCGG
		R	AAAGATATCAAAGTGCTATAAGATGCAC
	NCAM	F	TCACCCTGGTGTGCGATG
		R	ATGTACTTCTCATCGTCTTCCTCTTG
	NF68	F	GTGACCAAGCCCGACCTTT
		R	ATTCCTCAGCGTTCTGCATGT
	PRRX1	F	CATCGTACCTCGTCCTGCTC
		R	GAATCCGTTATGAAGCCCCT
	SNAI1	F	TTCTCACTGCCATGGAATTCC
		R	GCAGAGGACACAGAACCAGAAA
	TBP	F	GCCCGAAACGCCGAATAT
		R	CCGTGGTTCGTGGCTCTCT
	VIM	F	CTGCCAACCGGAACAATGA
		R	GTACTCAGTGGACTCCTGCTTT

F = forward, R = Reverse
